# A novel molecular diagnostics platform for somatic and germline precision oncology

**DOI:** 10.1002/mgg3.291

**Published:** 2017-04-23

**Authors:** Rubén Cabanillas, Marta Diñeiro, David Castillo, Patricia C. Pruneda, Cristina Penas, Guadalupe A. Cifuentes, Álvaro de Vicente, Noelia S. Durán, Rebeca Álvarez, Gonzalo R. Ordóñez, Juan Cadiñanos

**Affiliations:** ^1^ Instituto de Medicina Oncológica y Molecular de Asturias (IMOMA) S. A. Avda. Richard Grandío s/n 33193 Oviedo Spain; ^2^ Disease Research And Medicine (DREAMgenics) S. L. Vivero Empresarial de Ciencias de la Salud C/Colegio Santo Domingo de Guzmán s/n 33011 Oviedo Spain

**Keywords:** Cancer, counseling, diagnostics, NGS, targeted‐therapy

## Abstract

**Background:**

Next‐generation sequencing (NGS) opens new options in clinical oncology, from therapy selection to genetic counseling. However, realization of this potential not only requires succeeding in the bioinformatics and interpretation of the results, but also in their integration into the clinical practice. We have developed a novel NGS diagnostic platform aimed at detecting (1) somatic genomic alterations associated with the response to approved targeted cancer therapies and (2) germline mutations predisposing to hereditary malignancies.

**Methods:**

Next‐generation sequencing libraries enriched in the exons of 215 cancer genes (97 for therapy selection and 148 for predisposition, with 30 informative for both applications), as well as selected introns from 17 genes involved in drug‐related rearrangements, were prepared from 39 tumors (paraffin‐embedded tissues/cytologies), 36 germline samples (blood) and 10 cell lines using hybrid capture. Analysis of NGS results was performed with specifically developed bioinformatics pipelines.

**Results:**

The platform detects single‐nucleotide variants (SNVs) and insertions/deletions (indels) with sensitivity and specificity >99.5% (allelic frequency ≥0.1), as well as copy‐number variants (CNVs) and rearrangements. Somatic testing identified tailored approved targeted drugs in 35/39 tumors (89.74%), showing a diagnostic yield comparable to that of leading commercial platforms. A somatic *EGFR* p.E746_S752delinsA mutation in a mediastinal metastasis from a breast cancer prompted its anatomopathologic reassessment, its definite reclassification as a lung cancer and its treatment with gefitinib (partial response sustained for 15 months). Testing of 36 germline samples identified two pathogenic mutations (in *CDKN2A* and *BRCA2*). We propose a strategy for interpretation and reporting of results adaptable to the aim of the request, the availability of tumor and/or normal samples and the scope of the informed consent.

**Conclusion:**

With an adequate methodology, it is possible to translate to the clinical practice the latest advances in precision oncology, integrating under the same platform the identification of somatic and germline genomic alterations.

## Introduction

Precision oncology is at the frontline of personalized medicine. The use of a patient's molecular data to inform diagnosis, prognosis, treatment and prevention of cancer for that very patient is a paradigm changing approach. To drive the adoption of personalized cancer medicine, the integration of validated next‐generation sequencing (NGS) diagnostics platforms into clinical practice is essential.

Targeted therapies aimed at tackling specific genomic alterations, such as the tyrosine kinase inhibitor (TKI) imatinib or the monoclonal antibody trastuzumab, were initially approved exclusively for the treatment of one type of cancer (chronic myelogenous leukemia with the *BCR*‐*ABL* (MIM: 151410 and MIM: 200100, respectively) translocation and breast cancer with *ERBB2* (MIM: 164870) amplification, respectively) but they were also later shown to be effective on other tumor types with identical or similar alterations (gastrointestinal stromal tumors with *KIT* (MIM: 164920) or *PDGFRA* (MIM: 173490) mutations and gastric cancer with *ERBB2* amplification, respectively). This supports the application of a genomic‐driven transversal view of cancer therapy. The encouraging results of molecularly driven clinical trials, such as the so called “basket trials”, and case reports of exceptional responders to targeted therapies selected on the basis of somatic mutations, irrespective of primary tumor site or histology, are proving the utility of this transversal approach (Wagle et al. 2014; Hyman et al. 2015; Redig and Janne 2015).

For some cancer types, such as Non‐Small Cell Lung Cancer (NSCLC), several targeted drugs acting on different mutant proteins are approved by the FDA/EMA (TKIs acting on sensitive mutant EGFR proteins or ALK inhibitors directed against the products of *ALK* (MIM: 105590) translocations) and exploring additional therapeutic options with medicines approved for different indications is recommended by the NCCN guidelines (crizotinib or cabozantinib/vandetanib for NSCLC with *ROS1* (MIM: 165020) or *RET* (MIM:164761) translocations, respectively). In such cases, probing all cancer treatment options for a given patient with conventional, “first generation” genotyping approaches (PCR, Sanger sequencing, FISH, etc.) requires analyzing multiple samples from limited specimens, with different techniques and, in some cases, by different laboratories. This approach, which leads to rising costs and increasing turnaround times, is not suitable for the current scenario of ever‐growing targeted treatment opportunities (Iorio et al. 2016). However, NGS technologies, which allow analysis of multiple cancer biomarkers, including SNVs, indels, translocations and CNVs, on the same sample in a single assay, come as an optimal solution for this situation.

Although most actionable genomic alterations are acquired somatically, in some instances germline mutations are responsible for an inheritable increased risk for cancer development. Being able to identify these alterations is of utmost importance for providing patients with a comprehensive personalized clinical management. First, some germline alterations (such as those inactivating *BRCA1*/*BRCA2* (MIM: 113705 and MIM: 600185, respectively) or activating *RET*) are also linked to the response to targeted drugs. Moreover, when germline cancer predisposing mutations are identified, this facilitates prevention and early detection of future tumors both in the probands and in their families. Furthermore, analyzing matched germline DNA in parallel to the tumor DNA allows increased performance of the bioinformatics processing of NGS results. Because managing the request of a germline analysis and its clinical application may not be straightforward for oncology practitioners (as it involves specific genetic counseling, complex informed consent and, frequently, dealing with variants of uncertain clinical significance), the implementation of mechanisms to overcome these difficulties is especially welcome.

In this work, we present the development, validation and clinical application of a novel molecular diagnostic platform based on targeted NGS, specifically designed to comprehensively identify somatic alterations linked to sensitivity or resistance to approved cancer therapies and germline mutations predisposing to familial cancer. Moreover, we propose a results‐processing pipeline and an interpretation algorithm designed to deal with all different request situations, from exclusively somatic testing in the absence of germline DNA to combined somatic and germline analyses.

## Materials and Methods

### Ethical compliance

This study was approved by the competent ethics commitee (Comité Ético de Investigación Clínica Regional de1 Principado de Asturias).

### Patients

The study was performed on a consecutive series of unselected patients attending our clinic between December 2013 and February 2017. Patients involved in the somatic analysis were stage IV cancer patients who had progressed to first‐line chemotherapy. Patients involved in the germline analysis were all eligible for cancer genetic counseling based on their personal and/or familial history of cancer.

### Configuration of gene panels

#### Somatic subpanel

Identification of cancer genes with clinically relevant somatic mutations was based on searches in the COSMIC database (Forbes et al. 2015) (http://cancer.sanger.ac.uk/), mycancergenome.com (http://www.mycancergenome.com), targetedcancercare.com (http://www.targetedcancercare.com), Google Scholar and PubMed. As a result of those searches, an important source of information for building our somatic database was a comprehensive publication by Dienstmann and coworkers (Dienstmann et al. 2014). Next, the preliminary gene list was further filtered considering information contained in the FDA (www.fda.gov) and EMA (www.ema.europa.eu/ema/) web‐pages to identify those genes therapeutically relevant whose genomic alterations have been related to approved cancer therapies.

#### Germline subpanel

An initial list of cancer genes with germline alterations associated with hereditary cancer predisposition was generated through automatic searches on the HGMD database. Then, manual review of the preliminary gene list was performed based on OMIM (www.omim.org), Genetics Home Reference (ghr.nlm.nih.gov), Orphanet (www.orpha.net) and other sources with the aim of filtering out of the list those genes fulfilling any of the following criteria: (1) insufficient scientific evidence or HGMD annotation error; (2) mutations assigned to the gene in HGMD correspond to a genomic region not functionally characterized and/or (3) cancer‐associated alterations that may not be detected by the proposed methodology (uncharacterized gene fusions, inversions, translocations or epigenetic changes).

### GenBank accession numbers

For all genes, variants were annotated according to all the GenBank RefSeq transcripts used by HGMD for that gene (HGMD transcripts). Additionally, for those genes of the somatic subpanel, variants were also annotated according to the most prioritary APPRIS transcript listed by Ensembl, unless this coincided with an HGMD transcript. The RefSeq transcript GenBank Accession Numbers were: *ABL1* NM_007313.2; *AKT1* NM_005163.2; *ALK* NM_004304.4; *APC* NM_000038.5; *AR* NM_000044.3; *ARA* NM_001654.4, NM_001256196.1; *ARID1A* NM_006015.4; *ATM* NM_000051.3; *ATP2A2* NM_001681.3, NM_170665.3; *ATR* NM_001184.3; *AXIN1* NM_003502.3; *AXIN2* NM_004655.3; *BAP1* NM_004656.3; *BARD1* NM_000465.2; *BCR* NM_004327.3; *BLM* NM_000057.2; *BMPR1A* NM_004329.2; *BRAF* NM_004333.4; *BRCA1* NM_007294.3, NM_007300.3; *BRCA2* NM_000059.3; *BRIP1* NM_032043.2; *BTK* NM_000061.2; *BUB1B* NM_001211.5; *CBL* NM_005188.3; *CCND1* NM_053056.2; *CCND2* NM_001759.3; *CCND3* NM_001760.3; *CD274* NM_014143.3; *CDC73* NM_024529.4; *CDH1* NM_004360.3; *CDK4* NM_000075.3; *CDK6* NM_001259.6; *CDKN1A* NM_078467.2; *CDKN1B* NM_004064.3; *CDKN1C* NM_000076.2; *CDKN2A* NM_000077.4, NM_058197.4, NM_058195.3; *CDKN2B* NM_004936.3; *CDKN2C* NM_001262.2; *CEBPA* NM_004364.3; *CHEK2* NM_007194.3; *CRKL* NM_005207.3; *CSF1R* NM_005211.3; *CSF3R* NM_000760.3; *CTNNA1* NM_001903.2; *CTNNB1* NM_001904.3; *CTR9* NM_014633.3; *CTRC* NM_007272.2; *CYLD* NM_015247.2; *DAPK1* NM_004938.2; *DDB2* NM_000107.2; *DDR2* NM_006182.2; *DICER1* NM_177438.2; *DIS3L2* NM_152383.4; *DKC1* NM_001363.3; *DLEU7* NM_198989.2; *DNAAF1* NM_178452.4; *EGFR* NM_005228.3, NM_001346941.1 (*EGFRvIII*); *EGLN1* NM_022051.2; *EGLN2* NM_080732.3; *ENG* NM_000118.3; *EPAS1* NM_001430.4; *EPCAM* NM_002354.2; *EPHA2* NM_004431.3; *EPHB2* NM_017449.3; *EPHX1* NM_000120.3; *ERBB2* NM_004448.2; *ERBB3* NM_001982.3; *ERBB4* NM_005235.2; *ERCC1* NM_202001.2; *ERCC2* NM_000400.3; *ERCC3* NM_000122.1; *ERCC4* NM_005236.2; *ERCC5* NM_000123.3; *EXT1* NM_000127.2; *EXT2* NM_207122.1; *FAH* NM_000137.2; *FAM175A* NM_139076.2; *FAN1* NM_014967.4; *FANCA* NM_000135.2; *FANCB* NM_001018113.1; *FANCC* NM_000136.2; *FANCD2* NM_033084.3; *FANCE* NM_021922.2; *FANCF* NM_022725.3; *FANCG* NM_004629.1; *FANCI* NM_001113378.1; *FANCL* NM_018062.3; *FANCM* NM_020937.2; *FAS* NM_000043.4; *FASLG* NM_000639.1; *FBXW7* NM_033632.3; *FGFR1* NM_023110.2; *FGFR2* NM_000141.4, NM_022970.3; *FGFR3* NM_000142.4; *FGFR4* NM_002011.3; *FH* NM_000143.3; *FLCN* NM_144997.5, NM_144606.5; *FLT3* NM_004119.2; *FRS2* NM_006654.4; *GALNT12* NM_024642.4; *GATA2* NM_032638.4; *GATA3* NM_001002295.1, NM_002051.2; *GNA11* NM_002067.2; *GNAQ* NM_002072.3; *GNAS* NM_000516.4, NM_016592.2, NM_080425.2; *GPC3* NM_004484.3; *GREM1* NM_013372.6; H19 NR_002196.1; *HDAC2* NM_001527.3; *HFE* NM_000410.3; *HGF* NM_000601.4; *HNF1A* NM_000545.5; *HNF1B* NM_000458.2; *HOXB13* NM_006361.5; *HOXD4* NM_014621.2; *HRAS* NM_005343.2; *IDH1* NM_005896.2; *IDH2* NM_002168.2; *IGF1R* NM_000875.3; *IGF2* NM_000612.4, NM_001007139.4, NM_001127598.1; *IL10RB* NM_000628.4; *IL7R* NM_002185.3; *INHBA* NM_002192.2; *INPP4B* NM_003866.2; *ITK* NM_005546.3; *JAK1* NM_002227.2; *JAK2* NM_004972.3; *JAK3* NM_000215.3; *KCNQ1OT1* NR_002728.3; *KDR* NM_002253.2; *KHDC3L* NM_001017361.2; *KIF1B* NM_015074.3, NM_183416.3; *KIT* NM_000222.2; *KMT2A* NM_001197104.1; *KMT2C* NM_170606.2; *KRAS* NM_004985.3, NM_033360.2; *LZTR1* NM_006767.3; *MAP2K1* NM_002755.3; *MAP2K2* NM_030662.3; *MAPK1* NM_002745.4; *MAX* NM_002382.4; *MC1R* NM_002386.3; *MDH2* NM_005918.2; *MEN1* NM_130799.2; *MET* NM_001127500.1; *MITF* NM_000248.3, NM_006722.2; *MLH1* NM_000249.3; *MNX1* NM_005515.3; *MPL* NM_005373.2; *MRE11A* NM_005591.3; *MSH2* NM_000251.2; *MSH6* NM_000179.2; *MSR1* NM_138715.2; *MTAP* NM_002451.3; *MTOR* NM_004958.3; *MUTYH* NM_001128425.1; *MYD88* NM_002468.4; *NBN* NM_002485.4; *NF1* NM_000267.3, NM_001042492.2; *NF2* NM_000268.3; *NFKBIZ* NM_031419.3; *NHP2* NM_017838.3; *NLRP7* NM_206828.3, NM_139176.3, NM_001127255.1; *NOP10* NM_018648.3; *NOTCH1* NM_017617.3; *NOTCH2* NM_024408.3; *NRAS* NM_002524.4; *NRG1* NM_013956.3, NM_013964.3, NM_013962.2, NM_013959.3; *NSD1* NM_022455.4; *NTHL1* NM_002528.5; *NTRK1* NM_001012331.1, NM_002529.3; *NTRK3* NM_001012338.2, NM_002530.3; *OGG1* NM_002542.5; *PALB2* NM_024675.3; *PAX5* NM_016734.2; *PBRM1* NM_018313.4; *PDE11A* NM_016953.3; *PDGFB* NM_002608.2; *PDGFRA* NM_006206.4; *PDPK1* NM_002613.4; *PHOX2B* NM_003924.3; *PIF1* NM_025049.2; *PIK3CA* NM_006218.2; *PIK3CB* NM_006219.2; *PIK3R1* NM_181523.2; *PIK3R2* NM_005027.3; *PLCG2* NM_002661.3; *PMS1* NM_000534.4; *PMS2* NM_000535.5; *POLD1* NM_002691.3; *POLE* NM_006231.2; *POLH* NM_006502.2; *POT1* NM_015450.2; *PPARG* NM_015869.4; *PRF1* NM_001083116.1; *PRKAR1A* NM_002734.4; *PRKCD* NM_006254.3; *PRKCH* NM_006255.3; *PRSS1* NM_002769.4; *PSMC3IP* NM_016556.3; *PTCH1* NM_000264.3, NM_001083602.1; *PTCH2* NM_003738.4; *PTEN* NM_000314.4; *PTPN11* NM_002834.3; *PTPRD* NM_002839.3; *RAC1* NM_018890.3, NM_006908.4; *RAD51C* NM_058216.2; *RAD51D* NM_002878.3; *RAF1* NM_002880.3; *RB1* NM_000321.2; *RECQL* NM_002907.3; *RECQL4* NM_004260.3; *RET* NM_020975.4; *RHBDF2* NM_024599.5; *RICTOR* NM_152756.3; *RIT1* NM_006912.5; *RNASEL* NM_021133.3; *ROS1* NM_002944.2; *RPL11* NM_000975.3; *RPL35A* NM_000996.2; *RPL5* NM_000969.3; *RPS19* NM_001022.3; *RPS24* NM_033022.3, NM_001142285.1; *RPS7* NM_001011.3; *RTEL1* NM_032957.4; *RUNX1* NM_001754.4; *SASH1* NM_015278.3; *SBDS* NM_016038.2; *SDHA* NM_004168.2; *SDHAF2* NM_017841.2; *SDHB* NM_003000.2; *SDHC* NM_003001.3, NM_001035511.1; *SDHD* NM_003002.3; *SEC23B* NM_006363.4; *SEMA4A* NM_022367.3; *SERPINA1* NM_000295.4; *SFXN4* NM_213649.1; *SH2B3* NM_005475.2; *SH2D1A* NM_002351.4; *SHOC2* NM_007373.3; *SLBP* NM_006527.2; *SLC25A13* NM_014251.2; *SLX4* NM_032444.2; *SMAD4* NM_005359.5; *SMAD9* NM_001127217.2; *SMARCA4* NM_001128849.1; *SMARCB1* NM_003073.3; *SMO* NM_005631.4; *SOCS1* NM_003745.1; *SOS1* NM_005633.3; *SOS2* NM_006939.2; *SPINK1* NM_003122.3; *SRC* NM_005417.4; *SRP72* NM_006947.3; *SRY* NM_003140.2; *STAG2* NM_001042749.1; *STIM1* NM_003156.3; *STK11* NM_000455.4; *SUFU* NM_016169.3; *TERC* NR_001566.1; *TERT* NM_198253.2; *TFE3* NM_006521.4; *TINF2* NM_001099274.1; *TMEM127* NM_017849.3; *TMPRSS2* NM_001135099.1, NM_005656.3; *TP53* NM_000546.5; *TRIM37* NM_015294.3; *TSC1* NM_000368.4; *TSC2* NM_000548.3; *UBE2T* NM_014176.3; *UNC13D* NM_199242.2; *UNC5C* NM_003728.3; *VEGFA* NM_001025366.2; *VHL* NM_000551.3; *WNT10A* NM_025216.2; *WRAP53* NM_018081.2; *WRN* NM_000553.4; *WT1* NM_024426.4; *XIAP* NM_001167.3; *XPA* NM_000380.3; *XPC* NM_004628.4; *XRCC2* NM_005431.1; *XRCC4* NM_022406.2; *YAP1* NM_001130145.2.

### Iterative optimization of probe design

Ensembl Biomart was used to extract the genomic coordinates of all exons and selected introns from the different transcripts of each gene contained in any of the panels. These coordinates were merged to obtain nonredundant unique regions and those noncoding untranslated regions (UTRs) with clinical relevance were added. After this, SureDesign (Agilent, Santa Clara, CA, USA), was used to customize SureSelect enrichment probes with the following parameters: tiling density 5×, no masking and balanced boosting. Based on previously available exome sequencing data, low coverage regions contained within the genes of interest were identified and specific additional probes were designed.

For probe optimization, we calculated callabilities (percentage of bases covered with a given base quality, mapping quality and read depth) for each region after sequencing of the first 16 samples using version 1 (v1) of the probe design. For those regions showing callabilities below 100% at a 100× depth in a MiSeq run, additional probes were designed and the concentration of the existing ones was increased. Briefly, in the second version of the design (v2) we reduced the probe density from 5× to 3× for 2672 regions with an excess of sequencing depth based on v1 results and increased the probe density from 5× to 10× on 478 regions with poor callability, keeping the total number of probes in the same range as in v1 (within the limit of SureDesign Tier 2: 57.500 total probes). A similar approach was followed up for the design of successive versions of the probe. Furthermore, regions whose low callability was due to low mapping qualities caused by the presence of homologous regions in the reference genome were identified. All calculations for which no probe design version is specified were performed using v4.

### DNA isolation and quantification

#### Tumor tissues/cells

Macro/microdissection of each sample was performed to increase nucleated tumor cell content to ≥20%, and 0.025–0.1 mm^3^ of tumor‐enriched FFPE tissue were deparaffinized and pretreated with 1M NaSCN for 6–12 h to reverse formaldehyde‐induced crosslinks (Hosein et al. 2013). After this, DNA extraction was performed using the QIAamp DNA micro kit (Qiagen, Valencia, CA, USA). *Blood—*2 mL of total peripheral blood was processed with the Flexigene DNA kit (Qiagen). *Saliva—*1 mL of saliva preserved in DanaSaliva collection kits was processed using the Danagene saliva kit (Danagen‐BioTed, Badalona, Spain). All isolated DNA samples were quantified by both spectrophotometry, using NanoDrop (Thermo‐Fisher Scientific, Waltham, MA, USA) (N: Nanodrop‐concentration), and fluorimetry, using the Qubit^®^ dsDNA HS and/or BR assay kits (Thermo‐Fisher Scientific) (Q: Qubit‐concentration).

### Library construction and capture

50–200 ng of dsDNA (according to Qubit fluorimetric quantification) were processed following the protocol recommended by the manufacturer of the capture probes: “Low Input Sureselect^XT^ Target Enrichment System for Illumina Paired‐End Sequencing Library (Agilent).”

### Sequencing

Indexed libraries were sequenced using Illumina MiSeq, Nextseq500 or HiSeq1500 next‐generation sequencers following the equipment's instructions. Paired‐end 75–100 bp reads were obtained. MiSeq and NextSeq500 sequencing was performed at IMEGEN (Valencia, Spain), whereas HiSeq1500 sequencing was done by HealthInCode (A Coruña, Spain).

### Processing of sequencing data and variant calling

Raw FASTQ files were first evaluated using quality control checks from FastQC (http://www.bioinformatics.babraham.ac.uk/projects/fastqc/), Trimmomatic was then employed for read trimming and filtering. After removing low quality bases, adapters and other technical sequences, each library was aligned to the human reference genome (GRCh37) using BWA‐mem (Li and Durbin 2010) generating sorted BAM files with SAMtools (Li et al. 2009). Reads from the same libraries were then merged, removing optical and PCR duplicates using Picard (http://broadinstitute.github.io/picard/). High sequencing coverage depths allowed maintenance of sufficient independent reads at each target position after this step. Those regions with <20 reads of base quality ≥10 and mapping quality ≥20 were considered as target‐sequence gaps.

For the identification of germline point mutations, VarScan2 analysis was performed (Koboldt et al. 2012) (filtering parameters: total read depth ≥6, mutated allele count ≥3, variant frequency ≥0.1, base quality ≥20, mapping quality ≥30). For the identification of somatic substitutions and indels, a variation of Sidrón algorithm which has previously been described (Puente et al. 2011) was used (filtering parameters: total read depth ≥6, mutated allele count ≥3, variant frequency ≥0.025, base quality ≥20, mapping quality ≥30). In the latter, mutations detected in the tumor sample are interrogated in the normal sample in order to define the somatic status of each variant. In both cases the workflow performs a subsequent analysis to filter out recurrent sequencing errors, which is based on documented sequencing biases associated to NGS platforms (Kwon et al. 2013; Wall et al. 2014) as well as on the identification of specific sequencing errors found to be artifacts in previously evaluated samples. Moreover, visual inspection of the alignment is also performed as a final step for all reported variants.

The process of variant calling includes an extra evaluation layer for variants present in provided data‐sources (COSMIC for somatic and HGMD for germline variants), regardless of the filtering thresholds (including the 0.025 minimum variant frequency otherwise required). Variants called according to these criteria are still evaluated by the filtering thresholds, but instead of being discarded when quality measures are not met, the variant is registered in a different output, facilitating further inspection. The objective of this added flexibility is the identification of relevant mutations that would otherwise be missed when cutting thresholds are not met. We have evidenced the benefits of this procedure by correctly identifying variants in the 2016 EMQN External Quality Assessment scheme that presented a variant frequency below 0.025.

Variants were annotated using several databases containing functional (Ensembl, CCDS, RefSeq, Pfam), populational (dbSNP, 1000 Genomes, ESP, ExAC) and disease‐related (COSMIC, ICGC, HGMD professional) information, as well as 11 scores from algorithms for prediction of the impact caused by nonsynonymous variants on the structure and function of the protein (SIFT (Kumar et al. 2009), PolyPhen2 (Adzhubei et al. 2010), PROVEAN (Choi et al. 2012), Mutation Assessor (Reva et al. 2011), Mutation Taster (Schwarz et al. 2014), LRT (Chun and Fay 2009), MetaLR, MetaSVM (Dong et al. 2015), FATHMM and FATHMM‐MKL (Shihab et al. 2014)), and 1 score (GERP++) for evolutionary conservation of the affected nucleotide (Davydov et al. 2010).

The detection of CNVs was performed by a modified version of the exome2cnv algorithm (Valdes‐Mas et al. 2012) incorporating a combination of read depth and allelic imbalance computations for copy number assessment. For individual samples the algorithm employs a background of pooled samples processed using the same capturing protocol and sequencing technology. When paired tumor‐normal samples are available, a parallel analysis is performed to reevaluate copy numbers and determine the somatic status of variants.

A customized algorithm was developed to identify intrachromosomal and interchromosomal rearrangements. For their identification, the workflow examines discordant read pairs, mapping to different chromosomes or to the same chromosome at an anomalous distance, as well as split‐reads, presenting soft‐clipped and/or hard‐clipped bases. This procedure permitted identifying breakpoint events at nucleotide precision. These strategies have shown to be accurate for targeted gene panel‐based next‐generation sequencing (Abel et al. 2014).

### Generation of reference and NGS data for evaluation of analytical performance: sensitivity, specificity, positive predictive value, and ability to detect translocations

For calculating sensitivity and specificity, 10 immortal lymphoblastoid cell lines corresponding to 10 individuals whose genomes/exomes had been sequenced by the 1000 Genomes and HapMap projects were obtained from the Coriell Institute: NA20298 (ASW), NA12872 (CEU), NA18570 (CHB), HG00320 (FIN), HG00110 (GBR), A18960 (JPT), NA19020 (LWK), NA19794 (MXL), HG00740 (PUR) and NA18486 (YRI). Cell lines were cultured according to the protocols provided by Coriell, their DNAs isolated and mixed in equimolecular amounts. An NGS library was prepared, captured using the custom probe and sequenced in a single MiSeq run. Variants were called as described in the previous section and the results compared to those expected according to the genomic information available for these cell lines.

For obtaining the positive predictive value of the test on FFPE tumor DNA, 82 SNVs and indels previously identified by the platform in two different tumors with allelic frequencies, estimated by NGS read count, ranging from 0.11 to 0.88, were evaluated by PCR and Sanger sequencing. All variants were analyzed by direct and reverse sequencing.

We used the same two tumors to calculate sensitivity and specificity of the test on DNA extracted from FFPE samples. First, we made three mixtures of pre‐captured libraries prepared with DNA from each of the tumors in different proportions: 10% tumor #1 library/90% tumor #2 library; 50% tumor #1 library/50% tumor #2 library; and 90% tumor #1 library/10% tumor #2 library. The resulting three library‐mixes were captured with the probe (v4) and sequenced in three MiSeq runs, and variants were identified. To compare the results with those expected based on a technology alternative to NGS, we used GeneChip Genome Wide Human SNP Arrays (Affymetrix), hybridized at CeGeN‐PRB2‐ISCIII (Santiago de Compostela, Spain), with the aim of identifying positive and negative variant calls from these two patients. To maximize the number of variants, instead of focusing on somatic variants from the two tumors mentioned above, we analyzed by SNP arrays germline DNA from the patients in which the tumors originated. These germline variants should also be present in the corresponding tumor DNA (unless loss of the variant allele has happened somatically). To be able to evaluate variants at different frequencies for sensitivity calculations, we focused on SNP array‐positive variants exclusively present in one of the two patients, so that all of the variants selected get diluted when the two libraries are mixed. Thus, we only considered those variants detected by SNP‐arrays in one patient and not detected in the other. Variants absent from both patients were considered as negatives for specificity calculations. All expected occurrences of the same variant in any of the library mixtures were taken into account for sensitivity calculations, whereas all expected absences were considered for specificity calculations. Finally, the ability of the NGS platform to correctly call 225 positive variants with frequencies ≥0.05, 165 positive variants with frequencies ≥0.10 and 678 negative variants, was determined.

To test the ability to detect translocations, we obtained from Horizon Discovery (Cambridge, UK) three paraffin‐embedded positive control glass slides carrying *ALK* (HD319), *RET* (HD640) and *ROS1* (HD615) translocations. Considering the frequency of the translocated allele estimated by the manufacturer for each positive control and the Qubit quantification, a single DNA mixture was prepared in which each translocation was estimated to be present with an allele frequency between 0.04 and 0.05. An NGS library was prepared, captured with SureSelect using the v4 probe and sequenced in a single MiSeq run.

### Use of variant databases

#### Somatic variants

Somatic variant analysis is based on the Knowledge Database described by Dienstmann et al. (2014), which is periodically updated. A team of cancer genomicists and clinicians provide expert feedback on the contents of the database, including detection of potential errors which are communicated to its curators. Additions, removals and edition of the entries of each version of the database are identified and subjected to analysis, to detect novel associations between FDA and/or EMA approved targeted cancer drugs and gene variants. If new variants affect genes not included in the current version of the gene‐panel, all exons and/or selected introns affected by the variants are added to the following version of the gene panel.

#### Germline variants

Germline analysis was supported by the HGMD professional and ClinVar databases. Interpretation of database information is manually reviewed in the context of patient's phenotype, after in‐depth analysis of the literature.

### Variant classification and reporting

#### Somatic variants

For the classification and reporting of therapeutically relevant variants, we followed a methodology based on that proposed by Dienstmann et al. (Dienstmann et al. 2014), but focused on variants with allele frequencies below 0.05 in all the population databases evaluated, namely dbSNP, ExAC, ESP and 1000 Genomes, and that fulfill any of the following conditions: (1) they were present as somatic mutations in the COSMIC and/or the International Cancer Genome Consortium (ICGC) databases, (2) they were predicted to have an impact on the sequence of the proteins encoded by the genes they affect. Variants were evaluated by in depth literature analysis and filtered so that only those predicted to have an effect on the function of the gene or protein which has been related to sensitivity/response or resistance/no response to an approved targeted cancer drug were reported as clinically relevant. The information contained in the report was selected to balance space limitations and clear and unambiguous communication to the clinical team with the most recent recommendations for classification and interpretation of therapeutically relevant somatic cancer variants (Ritter et al. 2016; Li et al. 2017). The level of evidence for somatic variants with therapeutic relevance is summarized in the report using the following terms: (1) “Approved/Rejected by FDA and/or EMA” in the context of a specific tumor, (2) Recorded in the “NCCN and/or CAP” guidelines, (3) “Advanced Clinical‐Trial”, referring to prospective clinical trials guided by genomic analyses, (4) “Preliminary Clinical‐Trial”, referring to non‐advanced clinical trials showing preliminary evidence of efficacy or lack of it (5) “Clinical case”, referring to individual cases with exceptional responses to targeted therapies in a specific genomic context and (6) “Preclinical”, referring to robust but preclinical data which are being explored in clinical trials. Somatic variants of unknown significance were also reported in the corresponding section (Fig. S1).

#### Germline variants

For the classification of germline variants related to hereditary cancer we defined the following three categories: (1) (Known) Pathogenic variants: those considered as disease‐causing mutations (DM) by HGMD and related to hereditary cancer which, under individual up‐to‐date review of the literature, were considered to be supported by solid scientific evidence, and which were absent or showed germline allelic frequencies below 0.05 in all the population databases evaluated, namely dbSNP, ExAC, ESP and 1000 Genomes; (2) Likely pathogenic variants: those fulfilling the following four conditions: (a) they were not considered as DM by HGMD, (b) they were strongly predicted to produce a drastic effect on the sequence of the protein (premature stop codon, loss of initiation ATG codon, aberrant splicing or frameshift), (c) they affected genes for which germline loss of function was associated with an increased risk of developing cancer, (d) they were absent or showed germline allelic frequencies below 0.01 in any of the population databases evaluated; (3) Uncertain clinical significance variants: those fulfilling any of the following conditions: (a) they were not considered pathogenic, causal of hereditary cancer, by HGMD, nor likely pathogenic, and they showed a maximum allelic frequency below 0.01 in the considered population databases; or (b) they were considered pathogenic mutations and related to hereditary cancer (DM: disease‐causing mutation) by HGMD and showed a maximum allelic frequency below 0.05 in the considered population databases, but their association with cancer was based on insufficient and/or controversial evidence; or (c) they fulfilled all the criteria of likely pathogenic variants except for their allelic frequency, which was between 0.01 and 0.05 in the population databases considered. Variants were reported as shown in Fig. S2 and S3.

## Results

### Definition of the ONCOgenics gene panel

#### Somatic gene subpanel (ONCOgenics *Tumor*™)

We generated a list of cancer genes whose alterations predict sensitivity or resistance to targeted drugs approved for the treatment of cancer, irrespective of its anatomical origin or histology. This panel includes all coding exons from 97 genes for detection of SNVs, indels and copy number variants (CNVs), as well as specific introns from 17 genes to detect selected rearrangements (Table 1). Cancer genes whose somatic alterations only have diagnostic and/or prognostic clinical value (i.e., with no predictive value) are not included in the somatic subpanel.

**Table 1:** Tumor gene subpanel (v6)

**Gene list for the detection of single‐nucleotide variants, insertions/deletions and copy number variants (97)**								
*ABL1*	*BRCA2*	*CDKN2B*	*ERBB4*	*GNA11*	*INPP4B*	*MPL*	*PIK3R2*	*SH2B3*
*AKT1*	*CBL*	*CDKN2C*	*FANCA*	*GNAQ*	*JAK1*	*MTOR*	*POLE*	*SMO*
*ALK*	*CCND1*	*CHEK2*	*FBXW7*	*GNAS*	*JAK2*	*MYD88*	*PRKCH*	*SOCS1*
*AR*	*CCND2*	*CSF1R*	*FGFR1*	*HDAC2*	*JAK3*	*NF1*	*PTCH1*	*SRC*
*ARAF*	*CCND3*	*CSF3R*	*FGFR2*	*HGF*	*KDR*	*NF2*	*PTEN*	*STAG2*
*ARID1A*	*CD274*	*CTNNB1*	*FGFR3*	*HRAS*	*KIT*	*NRAS*	*RAC1*	*STK11*
*ATM*	*CDK4*	*DDR2*	*FGFR4*	*IDH1*	*KRAS*	*PALB2*	*RAD51C*	*TSC1*
*ATR*	*CDK6*	*EGFR*	*FLCN*	*IDH2*	*MAP2K1*	*PDGFRA*	*RAF1*	*TSC2*
*BAP1*	*CDKN1A*	*EPHA2*	*FLT3*	*IGF1R*	*MAP2K2*	*PIK3CA*	*RET*	*VEGFA*
*BRAF*	*CDKN1B*	*ERBB2*	*FRS2*	*IGF2*	*MET*	*PIK3CB*	*RICTOR*	
*BRCA1*	*CDKN2A*	*ERBB3*	*GATA3*	*IL7R*	*MITF*	*PIK3R1*	*ROS1*	
**Gene list for the detection of rearrangements (17)**								
*ALK*	*BCR*	*BRAF*	*ERBB4*	*FGFR2*	*FGFR3*	*JAK2*	*MET*	*NRG1*
*NTRK1*	*PDGFB*	*PDGFRA*	*RAF1*	*RET*	*ROS1*	*TFE3*	*TMPRSS2*	

#### Germline gene subpanel (ONCOgenics *Germline*™)

We selected 148 genes with germline mutations consistently associated with an increased risk of developing cancer on a 2‐tier system (Table 2). Tier‐1 includes 72 genes considered as predominantly cancer‐predisposing, being cancer the initial presentation or a primary clinical feature of the syndrome. Genes within Tier‐1 are sub‐classified based on whether there exist established guidelines/recommendations for the management of patients with germline pathogenic variants in them (Tier‐1a), or not (Tier‐1b). Tier‐2 includes 76 genes associated with cancer in patients with overt mainly noncancerous syndromic features. Although both sets of genes are included in the panel, the latter are only considered reportable in the setting of a syndromic patient being analyzed, thus increasing the clinical sensitivity, while reducing the number of reported variants that are unrelated to the clinical manifestations of the patient.

**Table 2:** Germline gene subpanel (v6). Genes in bold represent those 35 Tier 1 genes for which there exist established guidelines/recommendations for management of patients with germline mutations (Tier 1a). The remaining Tier 1 genes form Tier 1b

**Tier‐1 genes (72)**								
***APC***	***BRIP1***	*FANCA*	*FANCL*	*MEN1*	*NF2*	*PTCH1*	***SDHA***	*SUFU*
***ATM***	***CDH1***	*FANCB*	*FANCM*	*MET*	*NTHL1*	*PTCH2*	***SDHAF2***	*TERT*
*AXIN2*	***CDK4***	*FANCC*	*FH*	***MLH1***	***PALB2***	***PTEN***	***SDHB***	***TMEM127***
*BAP1*	*CDKN1B*	*FANCD2*	*FLCN*	***MSH2***	*PDGFRA*	***RAD51C***	***SDHC***	***TP53***
*BARD1*	***CDKN2A***	*FANCE*	*GREM1*	***MSH6***	***PMS2***	***RAD51D***	***SDHD***	*TSC1*
***BMPR1A***	***CHEK2***	*FANCF*	*KIT*	***MUTYH***	***POLD1***	*RB1*	*SLX4*	*TSC2*
***BRCA1***	*EGFR*	*FANCG*	***MAX***	***NBN***	***POLE***	***RET***	***SMAD4***	*UBE2T*
***BRCA2***	*EPCAM*	*FANCI*	*MC1R*	***NF1***	*POT1*	*RHBDF2*	***STK11***	***VHL***
**Tier‐2 genes (76)**								
*ALK*	*DIS3L2*	*FAH*	*KHDC3L*	*PHOX2B*	*RPL11*	*SEC23B*	*SPINK1*	*WT1*
*BLM*	*DKC1*	*FAS*	*KRAS*	*POLH*	*RPL35A*	*SERPINA1*	*STIM1*	*XIAP*
*BRAF*	*ERCC1*	*FASLG*	*LZTR1*	*PRF1*	*RPL5*	*SH2D1A*	*TERC*	*XPA*
*BUB1B*	*ERCC2*	*GPC3*	*MNX1*	*PRKAR1A*	*RPS19*	*SHOC2*	*TINF2*	*XPC*
*CBL*	*ERCC3*	*H19*	*MTAP*	*PRSS1*	*RPS24*	*SLC25A13*	*TRIM37*	
*CDKN1C*	*ERCC4*	*HFE*	*NHP2*	*PTPN11*	*RPS7*	*SMARCA4*	*UNC13D*	* *
*CYLD*	*ERCC5*	*HRAS*	*NLRP7*	*RAF1*	*RTEL1*	*SMARCB1*	*WNT10A*	* *
*DDB2*	*EXT1*	*ITK*	*NOP10*	*RECQL4*	*RUNX1*	*SOS1*	*WRAP53*	* *
*DICER1*	*EXT2*	*KCNQ1OT1*	*NSD1*	*RIT1*	*SBDS*	*SOS2*	*WRN*	* *

#### Translational research subpanel

Sixty four additional cancer‐related genes not considered to be validated targets for approved therapies or not unambiguously associated with a hereditary cancer predisposition, but showing preliminary evidence for their potential relevance in oncology, are included. Coding exons and/or relevant non‐coding regions are targeted by the panel for research purposes only and their alterations are not clinically reported, but included in a separate variant list, if required (Table 3).

**Table 3:** Translational research subpanel (v6)

**Genes (64)**						
*ATP2A2*	*DLEU7*	*GALNT12*	*KMT2C*	*PAX5*	*PTPRD*	*UNC5C*
*AXIN1*	*DNAAF1*	*GATA2*	*MAPK1*	*PBRM1*	*RECQL*	*XRCC2*
*BTK*	*EGLN1*	*HNF1A*	*MDH2*	*PDE11A*	*RNASEL*	*XRCC4*
*CDC73*	*EGLN2*	*HNF1B*	*MRE11A*	*PDPK1*	*SASH1*	*YAP1*
*CEBPA*	*ENG*	*HOXB13*	*MSR1*	*PIF1*	*SEMA4A*	
*CRKL*	*EPAS1*	*HOXD4*	*NFKBIZ*	*PLCG2*	*SFXN4*	
*CTNNA1*	*EPHB2*	*IL10RB*	*NOTCH1*	*PMS1*	*SLBP*	
*CTR9*	*EPHX1*	*INHBA*	*NOTCH2*	*PPARG*	*SMAD9*	
*CTRC*	*FAM175A*	*KIF1B*	*NTRK3* a	*PRKCD*	*SRP72*	
*DAPK1*	*FAN1*	*KMT2A*	*OGG1*	*PSMC3IP*	*SRY*	

a Only specific introns to detect selected rearrangements.

### Improved probe performance through iterative re‐design

We initially developed a single custom SureSelect probe design targeting the genes in our panel (version 1 or v1) and tested its performance by generating libraries from tumor DNA extracted from formalin‐fixed paraffin‐embedded (FFPE) tumor tissue and germline DNA from a single patient. MiSeq sequencing of such libraries produced total callabilities (percentages of bases covered with a given base quality, mapping quality and read depth) above 99% at depths of 10 (DP10), 20 (DP20) and 50 (DP50) reads (Table S1).

However, we observed differences in the distribution of reads throughout the target regions. Thus, we detected 173 regions for which callability was poor (i.e. <100% at DP20), whereas 2672 regions showed 100% callabilities at DP500, from a total of 6055 regions. With the aim of evening the sequencing depth throughout all target regions, we redistributed probe densities in successive versions of the design. This produced a notable improvement of the callabilities at the poor regions from 22.40% (v1) to 91.15% (v2) at DP50 (Table S2), although at the cost of a slight decrease on total callabilities at DP50 and DP100 (Table S1). Further redistribution of probes in v3 and v4 of the design allowed recovering callabilities above 99% at DP10, DP20 and DP50 in all regions (Table S3).

### Sensitivity, specificity, and positive predictive value of the developed platform for point mutations and indels

We evaluated the analytical performance of the NGS platform on DNA isolated from both fresh cells and FFPE tissues. The test showed sensitivities and specificities on cell lines above 99% for the detection of the 2639 variants (2524 SNVs and 115 indels) contained within the target region and present at frequencies ≥0.05. Furthermore, sensitivity and specificity for the 1679 variants with frequencies ≥0.1 were both above 99.5% (Table 4). The performance of the analysis for detection of SNVs and indels on freshly isolated DNA was reproducible on DNA isolated from FFPE tumor tissues. The analytical positive predictive value was 100% based on 82 variants with frequencies 0.11–0.88 initially detected by the platform in those two tumors, all of which were later confirmed by PCR + Sanger (Table S4). Moreover, the sensitivity of the platform on three mixtures of libraries obtained from two FFPE tumors was >99% (223/225) for variants previously detected by SNP‐arrays and expected to be present in the mixtures with frequencies ≥0.05 and 100% (165/165) for those with expected frequencies ≥0.1, under >99.5% specificity conditions (Tables S5, S6).

**Table 4:** Sensitivity and specificity of the test on the mixture of cell lines

Frequency	Sensitivity	Specificity
≥0.05	99.17% (SNV 99.30%; indel 96.60%)	>99.5%
≥0.10	99.66% (SNV 99.76%; indel 97.67%)	>99.5%
>0.20	99.70% (SNV 99.80%; indel 96.40%)	>99.5%

### Performance at highly homologous regions

Genomic areas with high levels of homology to other parts of the genome represent challenging targets for conventional NGS: it may be very difficult or impossible to map short sequencing reads originating from those regions to the correct reference genome position. Mandelker et al. (2016) have recently generated an exome‐wide census with three different lists of problematic highly homologous regions affecting exon sequences: low‐stringency, high‐stringency and NGS‐dead zones. Low‐stringency and high‐stringency zones show high degrees of homology to other regions of the genome, but with 1–5 mismatches per 250 nt window. This should facilitate their sequencing using proper mapping quality filters and taking into account the information originating from paired‐end reads for mapping purposes. However, NGS‐dead zones are regions ≥250 nucleotides long identical to at least another genomic region, and NGS approaches using inserts <250 nt are unable to accurately sequence them.

We compared Mandelker's three lists with the target regions of our somatic and germline subpanels (v6) and *PMS2* (MIM: 600259) exon 15‐3′UTR was the only NGS‐dead zone in them. 8 additional *PMS2* exons were represented in the high‐stringency (4 exons) or low‐stringency (4 exons) lists (Table S7). Thus, we used *PMS2*, a relevant Lynch Syndrome gene for which our laboratory had previous experience in its analysis (Borrás et al. 2013), to test the developed NGS platform on highly homologous regions.

We focused on variants from five patients whose *PMS2* gene had been previously evaluated by Sanger sequencing after a long‐range (LR) PCR strategy designed to distinguish the gene from its pseudogenes (Clendenning et al. 2006; Vaughn et al. 2010; Borrás et al. 2013). When the NGS results were aligned to a whole‐genome reference sequence, all the variants outside exon 15‐3′UTR were correctly detected. However, four false negative calls were identified, affecting two variants within the exon 15‐3′UTR NGS‐dead zone (NM_000535.5: c.2466T>C and c.*92dupA, with two occurrences each) (Table S8 and Fig. S4).

In the specific case of *PMS2*, on top of the homology issue, gene conversion events between its 3′ end (exons 11, 12, 13, 14 and 15‐3′UTR) and that of *PMS2CL* have been documented (van der Klift et al. 2010; Vaughn et al. 2010). As a result, any variant potentially present in exons 11 to 15 and detected by approaches that do not make use of LR PCR might correspond to either the *PMS2* or the *PMS2CL* locus. Accordingly, a false positive call was detected in one sample (NM_000535.5: c.2466T>C) (Table S8).

To improve the sensitivity in those *PMS2* regions, we realigned the NGS results with a reference sequence containing the *PMS2* locus only. This permitted 100% sensitivity of true *PMS2* variants (17/17), although at the expense of specificity (Table S9). Thus, although this strategy can be used on NGS‐dead zones or regions affected by gene‐conversion, any clinically relevant mutation potentially identified must be confirmed by target‐specific methods (i.e., LR PCR).

These results indicate that the current platform is able to accurately detect variants in highly homologous regions outside Mandelker's NGS‐dead zone, as long as they are not affected by gene‐conversion events (i.e., outside *PMS2*). Thus, the performance of ONCOgenics is expected to be good on all other genes from the germline and/or somatic subpanels, whereas variants from exons 11–15 of *PMS2*, detectable by aligning NGS results to a reference sequence restricted to the *PMS2* locus, must be validated by alternative, gene‐specific approaches.

### Ability to detect translocations

We evaluated the ability of the test to detect translocations affecting *ALK*,* ROS1* and *RET* on a mixture of DNA obtained from three paraffin‐embedded positive control cell lines, which contained each translocation present in an allelic fraction between 0.04 and 0.05. Analysis by NGS using a novel algorithm developed *ad hoc* for rearrangement identification successfully detected all three translocations and mapped them to nucleotide resolution (Table S10). Notably, before data analysis, the bioinformaticians involved in this evaluation were unaware of the 5′ partners of the *ROS1* and *RET* translocations, as well as of the chromosomal coordinates of all six breakpoints.

### External quality assessment of analytical performance

We participated in the 2016 Oncogene Panel Testing External Quality Assessment scheme organized by the European Molecular Genetics Quality Network (EMQN) to independently assess the performance of the test. The scheme evaluated the analysis of eight established cancer genes with 30 mutations present in total in the four FFPE samples (one of which, sample #4, was mildly formalin compromised), with variant frequencies between 0.007 and 0.667 as per ddPCR quantitation (Table. S7). Detection of mutations with frequencies below 0.025 in these samples was based on the calling process supporting variants present in the COSMIC database described above (see Materials and Methods).

**Figure 1:**
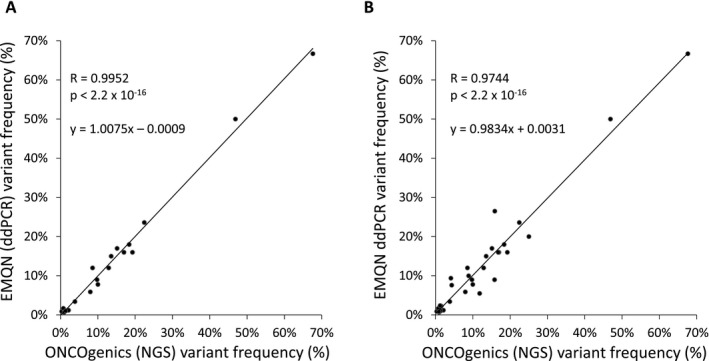
Correlation of variant frequencies detected by ddPCR and ONCOgenics. (A) Correlation between the frequencies of the variants present in three FFPE samples from the 2016 EQMN Oncogene Panel Testing External Quality Assessment Scheme as reported by EMQN (quantitated by ddPCR) and the frequencies of the same variants as determined by the NGS panel testing. (B) Same correlation as in (A) but after considering a fourth FFPE EMQN sample which had been mildly formalin compromised. The Pearson correlation coefficients (*R*), the *P*‐values (Pearson correlation test) and the linear regression equations (*y* = *ax *+ *b*) are shown.

ONCOgenics obtained the maximum score (2.00/2.00), as all present variants and no false positives were detected from all samples. Moreover, variant quantitation based on the NGS platform strongly correlated with the ddPCR variant frequencies reported by EMQN, even when considering the formalin compromised sample (*R* = 0.9952 for samples 1–3; *R* = 0.9744 for samples 1–4) (Fig. 1 and. Table 5).

**Table 5:** Performance of the platform on FFPE samples from the 2016 EMQN Oncogene Panel Testing External Quality Assessment Scheme. All variants were correctly detected. Percentages show the frequencies of the variants present as reported by EMQN (quantitated by ddPCR) and the frequencies of the same variants as determined by the developed NGS platform. Sample #4 was mildly formalin compromised. Analyses of these samples were performed with v6

Variants present in each sample	Estimated frequencies	
	EMQN (ddPCR) (%)	NGS platform (%)
**FFPE sample #1**		
*BRAF* (NM_004333.4) c.1799T>A; p.V600E	16	19.24
*KRAS* (NM_004985.3) c.38G>A; p.G13D	16	16.97
*PIK3CA* (NM_006218.2) c.3140A>G; p.H1047R	17	15.12
**FFPE sample #2**		
*BRAF* (NM_004333.4) c.1799T>A; p.V600E	66.70	67.62
*EGFR* (NM_005228.3) c.2155G>A; p.G719S	1.10	0.72
*EGFR* (NM_005228.3) c.2235_2249del15; p.E746_A750del	0.90	0.28
*EGFR* (NM_005228.3) c.2369C>T; p.T790M	1.20	2.11
*EGFR* (NM_005228.3) c.2573T>G; p.L858R	1.30	0.95
*EGFR* (NM_005228.3) c.2582T>A; p.L861Q	0.70	0.95
*PIK3CA* (NM_006218.2) c.3140A>G; p.H1047R	50	46.87
**FFPE sample #3**		
*BRAF* (NM_004333.4) c.1799T>A; p.V600E	12	12.88
*EGFR* (NM_005228.3) c.2155G>A; p.G719S	23.60	22.44
*EGFR* (NM_005228.3) c.2235_2249del15; p.E746_A750del	1.70	0.72
*EGFR* (NM_005228.3) c.2369C>T; p.T790M	1.02	1.33
*EGFR* (NM_005228.3) c.2573T>G; p.L858R	3.40	3.82
*KIT* (NM_000222.2) c.2447A>T; p.D816V	9	9.75
*KRAS* (NM_004985.3) c.35G>A; p.G12D	5.90	7.97
*KRAS* (NM_004985.3) c.38G>A; p.G13D	15	13.55
*NRAS* (NM_002524.4) c.181C>A; p.Q61K	12	8.55
*PIK3CA* (NM_006218.2) c.1633G>A; p.E545K	7.80	9.98
*PIK3CA* (NM_006218.2) c.3140A>G; p.H1047R	18	18.36
**FFPE sample #4 (formalin compromised)**		
*BRAF* (NM_004333.4) c.1799T>A; p.V600E	9.40	4.09
*EGFR* (NM_005228.3) c.2155G>A; p.G719S	26.50	15.87
*EGFR* (NM_005228.3) c.2235_2249del15; p.E746_A750del	2.30	1.50
*EGFR* (NM_005228.3) c.2369C>T; p.T790M	0.85	0.90
*EGFR* (NM_005228.3) c.2573T>G; p.L858R	2.40	1.20
*KIT* (NM_000222.2) c.2447A>T; p.D816V	9	15.79
*KRAS* (NM_004985.3) c.35G>A; p.G12D	5.50	11.81
*KRAS* (NM_004985.3) c.38G>A; p.G13D	16	16.79
*NRAS* (NM_002524.4) c.181C>A; p.Q61K	10	8.84
*PIK3CA* (NM_006218.2) c.1633G>A; p.E545K	7.60	4.32
*PIK3CA* (NM_006218.2) c.3140A>G; p.H1047R	20	25

### Determination of minimum sample requirements

According to the “Low Input Sureselect^XT^” library preparation protocol, 200 ng of double stranded DNA (dsDNA), measured by a method able to distinguish it from single stranded or degraded DNA (such as the Qubit fluorimetric assay), is the recommended amount of starting material. This is easy to obtain from blood or saliva samples, but may be a limitation for formalin‐fixed samples. Thus, based on our results with 46 formalin‐fixed samples, we determined the minimum DNA amount and quality requirements for successful library preparation (Table S11).

The Qubit‐concentration/NanoDrop‐concentration ratio (Q/N) is an indicator of DNA purity and/or quality, as spectrophotometric (NanoDrop) measurements are based on total absorbance at 260 nm whereas fluorimetric measurements are more specific for dsDNA. According to our series, a minimum of 100 ng of DNA with a Q/N higher than 0.29 is a good indicator of a DNA sample from FFPE tissue being of enough quality for library preparation. Nonetheless, samples with lower DNA amounts or Q/N ratios should not be discarded, as they allow library preparation most of the times. Q/N ratios lower than 0.1, however, predict library preparation failure or libraries with very low percentage of sequence on target (7/8 in our series).

Regarding the minimum tumor content of the sample, this is set at 20% because, according to Table 4, that would allow to detect, with >99.5% sensitivity and specificity, heterozygous somatic SNVs or indels present in 100% of the tumor cells (with a tumor content of 20% their allele frequencies would be ≥0.1).

Samples below the 20% tumor cellularity threshold can be enriched by macro/microdissection. However, we currently do not have a method for qualifying samples with Q/N lower than 0.1. In such cases, although library construction may be attempted, testing of a different sample is recommended.

Finally, in terms of tumor sample type, besides formalin‐fixed, paraffin‐embedded tissue obtained from excisional biopsies, we have also obtained good quality DNA and reliable results with small core biopsies and tumor cells from fine‐needle aspirates.

### Advantages of parallel tumor‐germline analysis

Analysis of a germline sample in parallel to the tumor sample is considered desirable by the most recent standards and guidelines for the interpretation of sequence variants in cancer (joint consensus recommendation of the Association for Molecular Pathology, American Society of Clinical Oncology, and College of American Pathologists) (Li et al. 2017). The relevance of matched germline testing for somatic mutation identification is threefold. First, during the processing of sequencing results, it allows removing systematic errors happening at low frequencies, which represent considerable noise for accurate identification of subclonal or diluted somatic variants, as previously demonstrated by us and others (Puente et al. 2011). Second, it allows unequivocal assignment of somatic versus germline variant origin (Schrader et al. 2016). Third, CNV identification is also benefited from this situation: in addition to the detection of differences with read distributions from previously sequenced samples, the analysis can be complemented with the comparison between paired tumor‐normal samples processed in parallel. These three advantages add up to improve the sensitivity and specificity of somatic variant identification.

To ascertain to which extent variants reported in established tumor‐only analyses are of germline origin, we evaluated the germline of 6 cases from our clinic previously sequenced by Foundation One™ (Frampton et al. 2013). As predicted, 21 out of 67 (31%) variants reported were of germline origin, and two of them had been linked to cancer therapies (Table S12). These results are in agreement with an independent systematic evaluation of tumor‐only versus paired tumor‐germline analysis of a targeted panel of 111 genes in 58 tumor samples, which concluded that 31% of the variants considered as somatic mutations by the tumor‐only analysis were actually of germline origin (Jones et al. 2015).

### Report structure

Reporting of clinical NGS findings has to be succinct and aimed at transmitting specifically the relevant results.

#### Somatic analysis

For the somatic analysis (request specifically oriented to the identification of therapeutic options for a cancer patient) we evaluate and report the genomic alterations related with sensitivity or resistance to approved cancer drugs according to the literature (for a more detailed description see “Materials and Methods” and Fig. S1).

#### Germline analysis

When a germline analysis is requested for identification of a cancer predisposing hereditary genomic alteration, we report the findings in three variant categories: pathogenic, likely pathogenic and variants with uncertain clinical significance (for a detailed description see “Materials and Methods” and Fig. S2). An “Additional comments” section summarizes pertinent negatives and includes mentions to potentially clinically relevant findings. Further detailed information is provided for each of the identified variants in the following report pages (Fig. S3).

Finally, both for the somatic and the germline analysis, information about the technical performance of the test on the sample analyzed is included in the report.

### Report modalities

**Figure 2:**
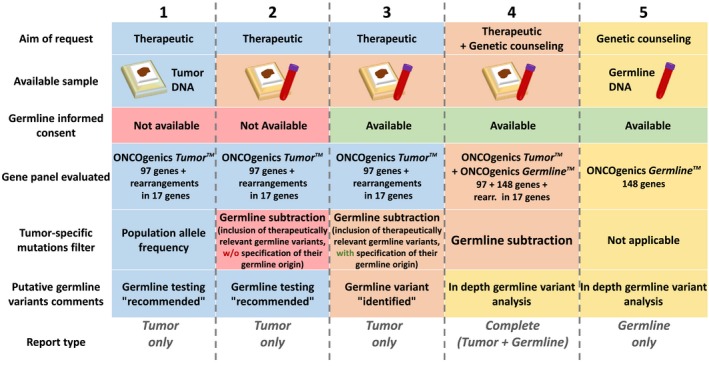
Graphical guide for adaptation of the sample‐to‐report pipeline. Columns 1 to 5 correspond to the different potential situations described in the main text. The first three rows (“Aim of request”, “Available sample” and “Germline informed consent”) represent the conditions which, combined, define those 5 situations. The last four rows (“Gene panel evaluated”, “Tumor specific mutations filter”, “Putative germline variants comments” and “Report type”), represent the combination of solutions taken to adapt the sample‐to‐report pipeline to each of those 5 situations.

When ordering tumor NGS testing, oncologists face complex situations to decide whether or not they should include parallel germline NGS testing, an issue which remains unsolved and under debate nowadays (Li et al. 2017). With the aim of fitting all possibilities, the report modalities of the described platform adapt to five different situations depending on the aim of the request, availability of tumor and/or germline sample and type of informed consent (Fig. 2):

1. the request has a therapeutic aim (finding approved cancer drug‐related genomic alterations) and only tumor sample is available;
2. the request has a therapeutic aim and both tumor and germline samples are provided, but no informed consent for reporting germline cancer predisposing mutations is available;
3. as in 2, but an informed consent is available which covers the disclosure of germline cancer predisposing mutations;
4. as in 3, but the request has both therapeutic and genetic counseling aims;
5. the request is aimed at genetic counseling, only germline sample is provided and an informed consent is available covering the disclosure of germline cancer‐predisposing mutations.

The algorithms and report modalities of the platform adapt to each of these situations:

In (1), those variants in the somatic gene panel most likely to be tumor‐specific are selected based on removing putative germline alterations frequently found in the population (those with variant frequency ≥0.05 in any of the four population databases considered: 1000 Genomes, ESP6500, ExAC and dbSNP). Genomic alterations below that threshold and related to approved cancer therapies according to the updated literature evidence are reported as clinically relevant. Additionally, if a non‐subclonal alteration is found which, when present in the germline, is known to cause hereditary cancer, a warning is included in the “Additional comments” section to call the clinician's attention so that the pertinent measures can be adopted (such as testing for the presence of the specific alteration on germline DNA after appropriate genetic counseling consultation).

In (2), we search for genomic alterations in our somatic gene panel related to available approved therapies in the tumor sequence, as in (1), but after bioinformatics removal of germline variants. In parallel, the germline sequence is also searched for variants with potential therapeutic relevance. This is done to avoid missing them as a result of the germline subtraction performed during the tumor analysis (i.e., a potential BRCA1 loss of function mutation present both in an ovarian cancer and in the germline sequence). We report all the therapeutically relevant variants as present in the tumor and, if any of them, not subclonal, is known to cause hereditary cancer when present in the germline, we include the same warning as in (1).

In (3), we perform the same kind of analysis as in (2), but we explicitly report the presence of hereditary cancer causing variants when they are found in the germline.

In (4), an analysis similar to that done in (3) is performed, but considering both the somatic and the whole germline gene subpanels. A report with two separate sections is issued, one section for the therapeutically relevant findings and the other with known or potential germline cancer‐predisposing variants (*Complete* report).

Finally, in (5) the germline panel is analyzed and the corresponding report is produced. Of note, when germline analyses are ordered, the requestors are given the option to restrict the report to Tier‐1a genes: those for which there exist established guidelines/recommendations for the management of patients with germline pathogenic variants.

### Clinical application

We evaluated the utility of the test in the clinical setting by applying it to 39 tumor and 36 germline samples from different patients.

#### Somatic findings

Analysis of the tumor samples revealed genomic alterations associated with approved cancer therapies in 89.74% of the analyzed cases (35/39) (Table 6). This yield is comparable to that reported in the literature (Zhong et al. 2015), and consistent with our previous experience with established platforms: analysis of 23 tumors from our clinic with Foundation One™ or Foundation One Heme™ had revealed approved therapies in 78% of the cases (18/23) (Table S13). Remarkably, all the somatic alterations identified by the Foundation tests associated with approved targeted therapies were in genes included in our somatic gene subpanel.

**Table 6:** Clinical performance of ONCOgenics *Tumor*™

Test ID	Tumor type	Actionable genomic alterations	Est. % tum. affect.	Associated drugs
ONCOE.001	Lung adenocarcinoma	*KRAS* c.35G>T; p.G12V	50–100	Everolimus, temsirolimus, trametinib
		*STK11* c.169G>T; p.E57*	50–100	Bosutinib, dasatinib, everolimus, temsirolimus, trametinib
ONCOE.002	Melanoma	*CDKN2A* c.143C>T; p.P48L	45–100	Palbociclib
ONCOE.003	Poorly diff. lung adenocarcinoma	None	NA	None
ONCOE.004	Undiff. renal carcinoma	None	NA	None
ONCOE.005	Lung adenocarcinoma	*EGFR* c.2573T>G; p.L858R	100	Afatinib, erlotinib, gefitinib
ONCOE.006	Thyroid anaplastic carcinoma	*PIK3CA* c.1624G>A; p.E542K	10–30	Everolimus, temsirolimus
		*NRAS* c.181C>A; p.Q61K	5–20	Trametinib
ONCOE.007	Laryngeal squamous cell carcinoma	*BAP1* c.1379C>G; p.S460*	95–100	Olaparib, valproic acid^a^
		*FGFR1* amplif.	NE	Pazopanib, ponatinib
ONCOE.008	Lung adenocarcinoma^b^	*EGFR* c.2237_2254del; p.E746_S752delinsA	75–100	Afatinib, afatinib + cetuximab, erlotinib, gefitinib,
ONCOE.009	Paraganglioma	None	NA	None
ONCOE.010	Thyroid medular carcinoma	*RET* c.2753T>C; p.M918T	50–100	Cabozantinib, sunitinib, vandetanib
		*EPHA2* c.1171G>A; p.G391R	15–40	Bosutinib, dasatinib, everolimus, temsirolimus
		*KRAS* c.35G>C; p.G12A	≤10	Everolimus/temsirolimus + trametinib,gemcitabine + trametinib, trametinib
ONCOE.011	Tongue squamous cell carcinoma	*EGFR* amplif.	NE	Cetuximab
ONCOE.012	Lung squamous carcinoma	*PTEN* c.802‐2A>T	25–60	Everolimus, olaparib, temsirolimus
		*CDKN2A* c.71G>C; p.R24P	20–50	Palbociclib
		*NF1* c.3299_3300del; p.A110Vfs4*	15–40	Everolimus, temsirolimus, trametinib
		*FGFR2* c.758C>G; p.P253R	5–20	Pazopanib
ONCOE.013	Primary peritoneal adenocarcinoma	*ERBB2* amplif.	NE	Ado‐trastuzumab, afatinib, emtansine, lapatinib, pertuzumab, trastuzumab
		*CDKN2A* c.172C>T; p.R58*	40–90	Palbociclib
ONCOE.014	Liposarcoma	*FRS2* amplif.	NE	Nintedanib, pazopanib, ponatinib
		*CDK4* amplif	NE	Palbociclib
ONCOE.015	Esophageal epidermoid carcinoma	*CDKN2A* c.176T>G; p.V59G	45–100	Palbociclib
ONCOE.017	Lung adenocarcinoma	*KRAS* c.183A>C; p.Q61H	30–70	Everolimus, palbociclib, temsirolimus, trametinib
		*STK11* c.157_158insG; p.D53Gfs*110	40–90	Bosutinib, dasatinib, everolimus, temsirolimus, trametinib
ONCOE.018	Colorectal adenocarcinoma	*CD274* amplif.	NE	Nivolumab, pembrolizumab
		*KRAS* c.35G>A; p.G12D	45–100	Cetuximab (no response), cobimetinib, palbociclib, panitumumab (no response), trametinib
ONCOE.019	Lung acinar adenocarcinoma	*FRS2* amplif.	NE	Nintedanib, pazopanib, ponatinib
		*NF1* deletion	NE	Everolimus, temsirolimus, trametinib
		*CCND1* amplif.	NE	Palbociclib
		*CDK4* amplif.	NE	Palbociclib
ONCOE.020	Mediastinal choriocarcinoma	*PTEN* c.328C>T; p.Q110*	45–100	Everolimus, olaparib, temsirolimus
ONCOE.021	Lung adenocarcinoma	*KRAS* c.37G>T; p.G13C	40–90	Everolimus, palbociclib, temsirolimus, trametinib
		*STK11* c.290 + 1G>C	45–100	Bosutinib, dasatinib, everolimus, temsirolimus, trametinib
ONCOE.023	Lymph node metastasis of adenocarcinoma of unknown primary site	*BAP1* deletion	100	Olaparib, platinum derivatives
		*BAP1* c.1769A>T; p.Q590L	100	
		*PALB2* deletion	100	Olaparib, platinum derivatives
		*PALB2* c.2590C>T; p.P864S	100	
		*FBXW7* deletion	100	Taxanes (resistance)
		*TSC1* deletion	100	Everolimus
		*TSC2* deletion	100	Everolimus
		*FLCN* deletion	100	Everolimus
		*PTCH1* deletion	100	Vismodegib
		*PTCH1* c.505G>A; p.V169I	100	
ONCOE.024	Lung adenocarcinoma	*NF1* c.7127‐1G>T	30–70	Everolimus, temsirolimus, trametinib
		*NF1* c.7395‐1G>T	30–70	
ONCOE.025	Glioblastoma multiforme	*EGFRvIII* variant (exons 2‐7 deletion)	NE	Erlotinib (no response)
		*CDKN2A* deletion	100	Palbociclib
		*CDKN2B* deletion	100	Palbociclib
ONCOE.026	Müllerian carcinosarcoma	None	NA	None
ONCOE.027	Lung adenocarcinoma with lepidic pattern	*KRAS* c.38G>A; p.G13D	15–40	Afatinib (no response), cobimetinib, erlotinib (no response), gefitinib (no response), palbociclib, trametinib
		*KRAS* c.34G>T; p.G12C	5–20	
		*ATM* c.8851‐2_8851‐1delAGinsTT	5–20	Olaparib
ONCOE.028	Germ cell ovarian tumor (Sertoli‐Leydig cell tumor)	*NF1* deletion	NE	Cobimetinib, everolimus, temsirolimus, trametinib
		*CTNNB1* c.110C>G; p.S37C	5–20	Everolimus + letrozole
		*CDK6* amplif.	NE	Palbociclib
ONCOE.029	Colorectal adenocarcinoma	*KRAS* c.35G>A; p.G12D	25–60	Cetuximab (no response), cobimetinib, palbociclib, panitumumab (no response), trametinib
ONCOE.030	Colorectal adenocarcinoma	*KRAS* c.35G>T; p.G12V	30–70	Cetuximab (no response), cobimetinib, palbociclib, panitumumab (no response), trametinib
ONCOE.031	PEComa	*PDGFRA* c.2526_2537del; p.I843_D846del	20–50	Imatinib, regorafenib, sunitinib
		*ATR* deletion	NE	Olaparib
ONCOE.032	Colorectal adenocarcinoma	*FLCN* deletion	NE	Everolimus, temsirolimus
		*PIK3R1* deletion	NE	Everolimus, temsirolimus
ONCOE.034	Small cell lung cancer	*PTEN* deletion	100	Everolimus, olpaparib, temsirolimus
		*PTEN* c.867dupA; p.V290Sfs*8	100	
		*BAP1* deletion	NE	Olaparib, panobinostat, valproic acid, vorinostat
		*BRCA2* deletion	NE	Nivolumab, olaparib, pembrolizumab
		*FLCN* deletion	NE	Everolimus, temsirolimus
		*PIK3R1* deletion	NE	Everolimus, temsirolimus
ONCOE.035	Colorectal adenocarcinoma	*BRAF* c.1799T>A; p.V600E	25–60	Cetuximab (no response), dabrafenib + trametinib, panitumumab (no response), vemurafenib (no response)
ONCOE.036	Nasal teratocarcinosarcoma	*CCND1* amplification	NE	Palbociclib
		*CDKN2A* deletion	NE	Palbociclib
		*CDKN2B* deletion	NE	Palbociclib
		*PALB2* c.1547delG; p.R516Kfs*45	10–30	Olaparib
ONCOE.039	Rectal adenocarcinoma	*NF1* c.204 + 1G>T	70–100	Everolimus, temsirolimus, trametinib, cobimetinib
ONCOT.040	Papillary thyroid cancer	*BRAF* c.1799T>A; p.V600E	45–100	Vemurafenib, dabrafenib, trametinib, cobimetinib, dabrafenib + trametinib
		*PIK3CA* c.1258T>C; p.C420R	25–60	Everolimus, temsirolimus
		*PIK3CA* c.3145G>C; p.G1049R	30–70	
ONCOT.041	Adenocarcinoma of unknown primary site (sample origin: pleura)	*EGFR* c.2573T>G; p.L858R (+ mutant allele amplification)	80–100	Erlotinib, afatinib, gefitinib
ONCOT.043	Ovarian serous papillary carcinoma	*NF1* c.320delC; p.T107Rfs*58	85–100	Everolimus, temsirolimus, trametinib, cobimetinib
ONCOT.044	Mesenteric fibromatosis	*CTNNB1* c.133T>C; p.S45P	30–70	Imatinib, everolimus + exemestane
ONCOC.001_T	Pancreatic adenocarcinoma	*FBXW7* c.1013_1016delGAAGinsAAA; p.R338Kfs*4	35–80	Everolimus, temsirolimus
		*FBXW7* c.1053G>A; p.W351*	35–80	
		*FBXW7* c.1095G>A; p.W365*	35–80	
		*KRAS* c.34G>T; p.G12C	45–100	Trametinib, cobimetinib, palbociclib

a Valproic acid is not an approved cancer drug, but it is a widely used and easily accessible HDAC inhibitor approved for the treatment of neurological disorders.

b Initial diagnosis: breast adenocarcinoma. Definitive diagnosis: lung adenocarcinoma (after pathology review motivated by NGS results).

Beyond its specific aims, comprehensive genomic profiling of the tumor can identify potential diagnostic errors. For instance, one of the analyzed tumors was labeled as an metastatic adenocarcinoma from a breast cancer, as it appeared as an isolated mediastinic lymph node, in a patient who had had a breast primary tumor 12 years before. However, after an *EGFR* (MIM: 131550) exon 19 indel was identified by ONCOgenics, pathology review was performed and immunohistochemistry revealed that it was a primary lung adenocarcinoma. Subsequently, the patient was treated with an EGFR tyrosin kinase inhibitor (gefitinib), obtaining a partial response, sustained to date, after 15 months on treatment.

#### Germline findings

Application of the test to 36 germline samples from individuals with a personal and/or family history of cancer identified a pathogenic genomic alteration in *CDKN2A* (MIM: 600160) (p.V59G) in a patient with familial melanoma, and a truncating *BRCA2* variant (p.A938Pfs*21) in a patient with familial breast/ovary cancer (Table 7). Genomic germline analysis also revealed unexpected clinically relevant findings such us the presence of a Xeroderma pigmentosum C recessive pathogenic alteration, *XPC* (MIM: 613208) (p.L763Cfs*) in heterozygosis in a healthy patient with a family history of breast/ovary cancer and leukemia, or a mosaic pathogenic mutation in *NF1* (MIM: 162200) (*NF1* p.I679Dfs*21, allelic frequency 10.3%) in another healthy patient with a family history of gastrointestinal cancer, prostate cancer and leukemia. This mosaic mutation, affecting a polyG tract within a low‐stringency highly homologous region, was validated by LR PCR followed by Sanger sequencing (Fig. S2 and Fig. S5).

**Table 7:** Clinical performance of ONCOgenics *Germline*™

Test ID	Pathogenic mutations	Likely pathogenic mutations	DM‐Controversial mutations	Other VUS
ONCOG.001	None	None	None	*ATM* c.998C>T; p.S333F (Het.)
				*CDH1* c.‐54G>C (Het.)
ONCOG.002	*CDKN2A* c.176T>G; p.V59G (Het.)	None	None	*BRCA2* c.4258G>T; p.D1420Y (Het.)
				*PTCH2* c.1073G>A; p.R358H (Het.)
				*SUFU* c.1018G>T; p.A340S (Het.)
ONCOG.003	None	None	None	*BRCA1* c.4883T>C; p.M1628T (Het.)
				*BRCA2* c.4258G>T; p.D1420Y (Het.)
				*FANCM* c.1667A>G; p.D556G (Het.)
				*PTCH2* c.1073G>A; p.R358H (Het.)
ONCOG.004	None	None	*BRCA2* c.7008‐62A>G (Het.)	*CDH1* c.‐54G>C (Het.)
				*SDHD* c.149A>G; p.H50R (Het.)
ONCOG.005	None	None	*FANCA* c.3348 + 18A>G (Het.)	None
ONCOG.006	None	None	None	*FANCA* c.932T>C; p.I311T (Het.)
				*FANCI* c.1573A>G; p.M525V (Het.)
				*MUTYH* c.1258C>A; p.L420M (Het.)
				*NF1* c.7259‐17C>T (Het.)
ONCOG.007	None	None	*FANCA* c.3348 + 18A>G (Het.)	*ATM* c.2119T>C; p.S707P (Het.)
				*AXIN2* c.1685C>T; p.P562L (Het.)
				*BRCA1* c.2890G>A; p.G964R (Het.)
				*FANCB* c.2395G>A; p.A799T (Het.)
				*FANCD2* c.3127G>A; p.G1043S (Het.)
ONCOG.008	None	None	None	*ATM* c.998C>T; p.S333F (Het.)
				*FANCM* c.5177C>T; p.P1726L (Het.)
ONCOG.009	None	None	*MC1R* c.464T>C; p.I155T (Het.)	*FANCD2* c.3275A>G; p.H1092R (Het.)
			*TSC2* c.5383C>T; p.R1795C (Het.)	*POLD1* c.189G>T; p.E63D (Het.)
				*SLX4* c.5501A>G; p.N1834S (Het.)
ONCOG.010	None	None	None	*FANCM* c.3857G>T; p.S1286I (Het.)
				*MSH6* c.*20_*24del (Het.)
ONCOG.011	None	None	*KIT* c.67 + 4G>A (Het.)	*PMS2* c.1688G>T; p.R563L (Het.)
				*SDHD* c.34G>A; p.G12S (Het.)
				*SUFU* c.1018G>T; p.A340S (Het.)
ONCOG.012	None	None	None	*MLH1* c.1852_1853delAAinsGC; p.K618A (Het.)
				*ATM* c.2519A>T; p.D840V (Het.)
				*FLCN* c.979G>A; p.A327T (Het.)
ONCOG.013	None	None	*ATM* c.1810C>T;p.P604S (Het.)	*ATM* c.6067G>A; p.G2023R (Het.)
ONCOG.014		None	*RET* c.1529C>T; p.A510V (Het.)	*MET* c.504G>T; p.E168D (Het.)
				*SDHD* c.34G>A; p.G12S (Het.)
			*FANCA* c.3348 + 18A>G (Het.)	*POLE* c.2090C>G; p.P697R (Het.)
				*BRCA1* c.3083G>A; p.R1028H (Het.)
ONCOG.015	None	None	None	None
ONCOG.016	None	None	*ATM* c.1744T>C; p.F582L (Het.)	*BARD1* c.2212A>G; p.I738V (Het.)
				*BMPR1A* c.478A>G; p.M160V (Het.)
				*CHEK2* c.254C>T; p.P85L (Het.)
ONCOG.017	None	None	None	*FANCF* c.557C>T; p.A186V (Het.)
				*PALB2* c.2590C>T; p.P864S (Het.)
				*POLD1* c.2317G>A; p.A773T (Het.)
				*SUFU* c.1018G>T; p.A340S (Het.)
ONCOG.018	None	None	None	None
ONCOG.019	None	None	*FANCA* c.3348 + 18A>G (Het.)	*BRIP1* c.584T>C; p.L195P (Het.)
				*SLX4* c.710G>A; p.R237Q (Het.)
				*SLX4* c.4597G>C; p.A1533P (Het.)
ONCOG.020	None	None	None	*ATM* c.8560C>T; p.R2854C (Het.)
				*EPCAM* c.488G>A; p.R163Q (Het.)
ONCOG.021	None	None	*RET* c.2531G>A p.R844Q (Het.)	*BARD1* c.2212A>G; p.I738V (Het.)
				*BRIP1* c.3275C>A; p.P1092Q (Het.)
				*MUTYH* c.950T>C; p.L317P (Het.)
ONCOG.022	None	None	None	*ATM* c.2572T>C; p.F858L (Het.)
				FANCA c.1870G>T; p.A624S (Het.)
				*FANCC* c.29G>A; p.C10Y (Het.)
				*PTCH2* c.1073G>A; p.R358H (Het.)
				*SDHD* c.34G>A; p.G12S (Het.)
ONCOG.023	None	*FANCM* c.4005dupA; p.V1336Sfs*8 (Het.)	None	*ATM* c.2119T>C; p.S707P (Het.)
				*BAP1* c.1769A>T; p.Q590L (Het.)
				*BRCA1* c.591C>T; p.C197C (Het.)
				*CDH1* c.532‐18C>T (Het.)
				*FANCC* c.77C>T; p.S26F (Het.)
				*PALB2* c.2590C>T; p.P864S (Het.)
				*PTCH1* c.505G>A; p.V169I (Het.)
ONCOG.024	*NF1* c.2033dupC; p.I679Dfs*21 (Mosaic)	None	None	*FANCA* c.3099C>A; p.D1033E (Het.)
				*BRCA1* c.3929C>A; p.T1310K (Het.)
ONCOG.025			*BARD1* c.1075_1095del; p.L359_P365del (Het.)	*FANCM* c.3857G>T; p.S1286I (Het.)
				*MC1R* c.252C>A; p.D84E (Het.)
			*cdkn2ai3* c.187G>C; p.G63R (Het.)	*PALB2* c.2590C>T; p.P864S (Het.)
				*PALB2* c.232G>A; p.V78I (Het.)
ONCOG.026	None	None	None	*FANCA* c.3099C>A; p.D1033E (Het.)
ONCOG.027	None	None	None	*BRCA1* c.3708T>G; p.N1236K (Het.)
				*FANCC* c.584A>T; p.D195V (Het.)
				*FANCI* c.3812C>T; p.S1271F (Het.)
				*FANCM* c.4931G>A; p.R1644Q (Het.)
				*MSH6* c.100G>T; p.A34S (Het.)
ONCOG.028	None	None	*SLX4* c.421G>T; p.G141W (Het.)	*BRCA2* c.68‐7T>A (Het.)
ONCOG.029	None	None	None	*MET* c.504G>T; p.E168D (Het.)
				*SDHD* c.149A>G; p.H50R (Het.)
ONCOG.030	None	None	*ATM* c.1810C>T; p.P604S (Het.)	*BRCA1* c.4039A>G; p.R1347G (Het.)
				*TSC2* c.1577G>A; p.S526N (Het.)
ONCOG.031	None	None	*FANCA* c.4069_4082del; p.A1357Lfs*63 (Het.)	*FANCM* c.171G>C; p.L57F (Het.)
				*RET* c.166C>A; p.L56M (Het.)
ONCOG.032	None	*FANCL* c.1051_1052delAG; p.S351Ffs*2 (Het.)	None	*BARD1* c.1028 C>T; p.T343I (Het.)
				*NBN* c.1591A>G; p.I531V (Het.)
				*PTCH1* c.1306G>A; p.D426N (Het.)
ONCOG.033	*BRCA2* c.2806_2809del; p.A938Pfs*21 (Het.)	None	*FANCA* c.1874G>C; p.C625S (Het.)	None
ONCOG.034	None	None	None	*APC* c.4424C>T; p.A1475V (Het.)
				*TMEM127* c.221A>G; p.Y74C (Het.)
ONCOG.035	None	None	None	*VHL* c.269A>G; p.N90S
				*APC* c.3949G>C; p.E1317Q
ONCOC.001_G	None	None	None	*SDHD* c.34G>A; p.G12S
				*SDHD* c.149A>G; p.H50R

We also detected several variants listed at the moment of clinical interpretation of results by HGMD as pathogenic (disease causing mutation or DM) but, in our view, with controversial or insufficient support in 41.67% of the cases (15/36; based on Tier‐1 genes only), which is in agreement with previous observations (Olfson et al. 2015; Groth et al. 2016). We classified these variants as “DM‐Controversial” and reported them under the “uncertain clinical significance variants” category, separated from the robust pathogenic variants.

## Discussion

The relevance and number of approved targeted cancer therapies is continuously growing. Most of them are approved for one or a very limited number of cancer histologies. However, some have demonstrated effectiveness against different tumor types as long as they harbor specific genomic alterations. Using NGS information for the selection of targeted therapies tailored to individual cancer cases is the basis of transversal precision oncology. The approach presented herein is especially suited for stage IV patients which have progressed to standard treatment options, when non‐standard therapy is considered. The platform is also adapted by design to situations in which germline predisposition to cancer development is in play, either suspected or not. The detection of germline genomic alterations that cause increased cancer risk allows to extend personalized patient management to prevention, early detection and genetic counseling.

Since insufficiently validated tests do represent a threat to patients, we have devoted a significant effort to demonstrate that the current platform is ready for clinical use. It provides reliable results on formalin‐fixed paraffin‐embedded tumor tissue, small core biopsies and fine‐needle aspirates, with diagnostic yields comparable to those of wider platforms and within a clinically useful timeframe (3 weeks for somatic analysis, 4 weeks for germline analysis). Additionally, the proposed single‐test design simplifies the laboratory workflow, facilitates scaling up, and represents a good alternative to running a series of multiple single‐gene companion tests on the often scarce tumor tissue. Indeed, parallelizing the analysis of multiple markers also maximizes the rate of actionable somatic findings (89.74% in our case). All this is in agreement with recent data on the analytical and the clinical utility of cancer gene panels, predicting that 80% of patients could benefit directly from tumor sequencing (Jones et al. 2015; Zhong et al. 2015).

The high sensitivity, specificity and positive predictive value of our test are consistent with the very good performance of the bioinformatics tools on which it is based, initially developed for the Chronic Lymphocytic Leukemia (CLL) genome project within the International Cancer Genome Consortium (ICGC). These tools have been extensively validated in the literature for the identification of clinically relevant somatic and germline mutations and have contributed for the CLL project to be the first in the ICGC to reach the 500 tumor/normal genome pairs goal (Puente et al. 2011, 2015; Quesada et al. 2012; Fanjul‐Fernández et al. 2013; Ramsay et al. 2013). Combining strong bioinformatics with high coverage (average of 950x), our platform is able to perform sensitive analysis of clonal heterogeneity, detecting mutant subclones which may be relevant for drug resistance.

Our analysis and reporting strategy is compatible with all options regarding sample availability, extension of informed consent and aim of request (Fig. 2). The proposed approach avoids potential problems generated by other strategies, such as missing a therapeutically actionable variant which is present both in the tumor and in the germline because of its subtraction by the bioinformatics analysis pipeline or failing to report a relevant hereditary cancer‐causing germline mutation when only tumor DNA is analyzed. Moreover, comprehensive germline analysis also provides the potential for unexpected identification of mutation carriers of well‐known cancer susceptibility syndromes in families with atypical phenotypes not meeting the established diagnostic criteria (LaDuca et al. 2014; Susswein et al. 2016). Future applications of NGS approaches, such as detection of cancer patients more likely to respond to immunotherapy, are also foreseeable (Le et al. 2015; Rizvi et al. 2015; Van Allen et al. 2015; Bouffet et al. 2016).

In spite of the great potential of NGS approaches for comprehensive mutational profiling of tumor and germline samples, there remain relevant challenges for which we propose a series of solutions (Gagan and Van Allen 2015):

### Selection of the gene panel and clinical implications

Our somatic approach, focused on genes whose alterations have been related to approved cancer therapies only, is aimed at making the most of the information contained in the tumor related to accessible targeted cancer drugs. In our experience, and in agreement with previous publications, the yield of clinically relevant findings is not negatively affected by focusing on a panel of genes when gene selection is carefully performed (Zhong et al. 2015). On the other hand, although there exist much smaller tissue‐specific panels with reduced cost compared to the presented approach, those are not suitable for centers interested in transversal precision oncology applications. We recognize that, when a promising tailored therapeutic option is found for a given cancer patient, unless it is approved for the very indication of the case, it is most of the times very hard and frequently impossible to get access to the drug, even for those approved for other indications (47, 48). However, hopefully, in the same way the design of clinical trials is changing, approval of commercialized medicines for novel cancer indications and access to off‐label drugs is also likely to adapt to the enormous possibilities of transversal oncology.

Regarding the germline analysis, most panels, as ours, include both high‐penetrance and moderate‐penetrance genes. This increases the chances of finding causal variants, at the price of raising the number of total variants found, many of them of uncertain clinical significance (VUS) (Lincoln et al. 2015). Performing careful interpretation of VUS is essential to avoid inappropriate changes on the clinical management of any patient based on the findings of an incorrectly evaluated variant. Additionally, by limiting germline reports to Tier1a genes, requestors have the option to restrict the analysis to those genes with the highest and clearest clinical actionability.

The use of a targeted strategy, as opposed to exome‐ or genome‐based approaches, while allowing more comprehensive and deeper coverages of the regions of interest (including introns for rearrangement identification which, in any case, are not covered by the exome), means that the panel needs to be redesigned and re‐validated as new knowledge about clinically relevant genomic alterations emerges. This is the reason why we keep in our probe design Tier‐3 genes, with suspected but, so far, not validated clinical utility and, thus, more likely to be found to be clinically relevant in the near future than the genes not included in the design at all. Moreover, the combination of genes with clinically relevant somatic and/or germline alterations in our panel also reduces the need for re‐design as, not rarely, a gene previously known as a target for clinically relevant somatic alterations is found to be a novel hereditary cancer gene and *vice versa*.

### Target‐sequence gaps

We have improved the performance and evenness of our capture‐probe in a way that the fraction of insufficiently covered target regions is minimized. Currently, the average fraction of insufficiently covered target‐sequence in our somatic panel is 0.043% (ranging from 0.0002% to 0.11%; *n* = 13), with an average sequencing depth of 941‐fold (ranging from 353‐fold to 1630‐fold). Regarding the germline panel, the average fraction of insufficiently covered target sequence is 0.016% (ranging from 0% to 0.1%; *n* = 9), with an average sequencing depth of 959‐fold (ranging from 265‐fold to 1534‐fold). Thus, we find it unnecessary to complete the analysis with Sanger sequencing. Alternatively, we provide a detailed list of the scarce insufficiently covered positions in the report of each case (Fig. S1).

### Results verification

Current recommendations advise Sanger verification of clinically relevant variants identified by NGS (Matthijs et al. 2016; Susswein et al. 2016). In the case of the current test, with sensitivities and specificities above 99.5%, we consider that verification of SNVs and indels might be skipped, specially for the somatic analysis, when the time window for clinical application of the results is very narrow. For the germline analysis, complementary approaches are likely to provide more accurate results for certain variant types (i.e., exon‐focused CGH‐arrays for single‐exon CNV detection or LR PCR‐based analyses for target genes with high degree of homology to other parts of the genome, such as *PMS2*) (Gray et al. 2015; Retterer et al. 2015).

### Clinical interpretation

In our series of 36 cases analyzed by our germline subpanel and considering only genes in Tier‐1, 15 cases (41.67%) had variants that were classified as disease causing mutations by HGMD but were degraded to DM‐Controversial after expert review. This is in agreement with previous reports (Olfson et al. 2015; Groth et al. 2016). We recommend performing clinical interpretation by carefully reviewing those publications supporting the HGMD and/or ClinVar classifications. When this is not conclusive, we recommend completing the analysis by searching available publications in PubMed and Google Scholar and following the recommendations provided by the ACMG/CAP guidelines for variant classification (Richards et al. 2015).

An additional difficulty for clinical interpretation is dealing with germline pathogenic/likely pathogenic variants in genes for which the risks associated to deleterious genetic alterations are not firmly established. We distinguish between two different situations:

- Pathogenic/Likely pathogenic variants affecting genes with associated cancer risks matching the personal/family history of the proband: the presence of the variant can be considered as an additional risk factor to tailor the proband's cancer surveillance program (which should be designed on a case by case basis, under the light of the cancer history of the proband and the family).
- Pathogenic/Likely pathogenic variants affecting genes with associated cancer risks not matching the personal/family history of the proband: the variant can be considered as a variant of uncertain significance and, thus, not taken into account.

### Challenging reporting process

Our reporting strategy shows all the clinically relevant information in the first page of each report with the aim of optimizing data visualization and adapting to the situation and preferences of each patient/requestor (Figs. 2 and S1, S2). For the somatic analysis, this involves displaying the identified alterations together with their associated therapeutic options at first sight, followed by the scientific literature supporting those associations. For the germline analysis, it involves clearly identifying pathogenic, likely pathogenic and uncertain significant variants on the front page, offering a detailed description of each variant in the subsequent pages. A sufficiently explicit technical information (including the analyzed genes and the performance of the test) is also essential for the clinician to understand the relevance of both positive and negative results.

## Conclusions

Clinical application of precision oncology is a reality. However, we are still taking the first steps, and difficulties, even beyond the actual NGS testing and reporting procedure, are evident. Platforms such as ONCOgenics are examples of the core building blocks that will be necessary to scale up the field, able to help clinicians, patients and families in the meanwhile of this transformation process. As proven by the genomic alterations explaining certain cases of exceptional responders, the potential for any given cancer patient to benefit from precision medicine is not just speculative or something to consider for the future, but evident and for the present.

## Conflicts of Interests

The following authors are currently employed by IMOMA or DREAMgenics, the companies involved in the development and exploitation of the ONCOgenics™ platform: IMOMA: R.C. (Physician in Chief), M.D. (Clinical Molecular Geneticist), G.A.C. (Biotechnologist), N.S.D. (Lab. Technician), R.A. (Lab. Technician) and J.C. (Lab. Director); DREAMgenics: D.C. (Bioinformatitian), P.C.P. (Bioinformatitian) and G.R.O. (Bioinformatitian, C.S.O. and C.E.O.).

## Supporting information


**Figure S1.** Front page of the somatic sample report.

**Figure S2.** Front page of the germline sample report.

**Figure S3.** Detailed information on germline variants.

**Figure S4.** Causes of false negative calling of variants in highly homologous *PMS2* regions.

**Figure S5.** Sanger validation of the mosaic *NF1* c.2033dupC mutation.

**Table S1.** Total callabilities in ONCOgenics v1 and v2.
**Table S2.** Callabilities at “poor” regions in ONCOgenics v1 and v2.
**Table S3.** Total callabilities in ONCOgenics v3 and v4.
**Table S4.** Variants detected in tumor #1 (white background) and tumor #2 (gray background). All the detected variants were validated by Sanger sequencing (PPV = 100%; 82/82).
**Table S5.** Variants previously identified by SNP‐arrays in two patients and used to calculate the sensitivity of the platform on mixtures of two tumors originated from the same patients.
**Table S6.** Positions previously identified as negative by SNP‐arrays in two patients and used to calculate the specificity of the platform on mixtures of two tumors originated from the same patients.
**Table S7.** High homology regions affecting PMS2 exons (as per Mandelker et al.).
**Table S8.** PMS2 variants called after aligning ONCOgenics *Germline*™ results against the whole reference genome.
**Table S9.** Potential PMS2 variants called in the exon 11‐3′UTR interval after aligning ONCOgenics *Germline* ^TM^ results against the PMS2 locus alone.
**Table S10.** ALK, ROS1 and RET translocations analyzed by ONCOgenics v4.
**Table S11.** Q/N ratio of libraries with DNA extracted from formaldehyde‐fixed specimens.
**Table S12.** True somatic versus germline origin of 68 variants reported in 6 Foundation One tests.
**Table S13.** Somatic alterations associated with approved targeted cancer therapies reported by Foundation One/One Heme tests performed on samples from 23 patients of our clinic.
