# Supplementary material for: A novel molecular diagnostics platform for somatic and germline precision oncology
Source: Mol Genet Genomic Med. 2017 Apr 23;5(4):336–59. doi: 10.1002/mgg3.291 (PMC5511795; doi:10.1002/mgg3.291)
Supplement: Supplementary file 1 — Figure S1. Front page of the somatic sample report. [file MGG3-5-336-s001.pdf]

Sample Tumor Report

I

GENOMIC ALTERATIONS DETECTED WITH THERAPEUTIC IMPLICATIONS

Gene	Reference transcript	Variant	Variant consequence (molecular)	Somatic status	% Tum. cel. mut.	Drugs approved in the analyzed tumor	Drugs approved in other tumors
PTEN	NM_000314	c.867dupA; p.Val290Serfs*8	Frameshift / Premature STOP	Inferred somatic	100%	Everolimus (FDA, no EMA)	Everolimus, temsirolimus, olaparib
	NM_000314	Deletion	Copy number loss (n=1)				
BAP1	NM_004656	Deletion	Copy number loss (n=1)	Inferred somatic	Not applicable	None	Olaparib, valproic acid, vorinostat and panobinostat

No actionable alterations have been found in the following **genes relevant** in **lung tumors**: ALK, ARAF, BRAF, DDR2, EGFR, EPHA2, ERBB2, ERBB4, FGFR1, FGFR2, IGF1R, KRAS, MET, KMT2D, NRAS, NRG1, NTRK1, PIK3CA, RET, RICTOR, ROS1, STK11.

II

SCIENTIFIC AND MEDICAL EVIDENCE SUPPORTING THE ASSOCIATIONS BETWEEN GENOMIC ALTERATIONS AND DRUGS

Variant	Therapy	Tumor type	Effect	Level of evidence[3]	References (PMID, abstract)
PTEN c.867dupA; p.Val290Serfs*8 + Deletion (n=1)	PI3K pathway inhibitors	Lung cancer	Sensitivity	Preclinical	23136191
		Prostate cancer	Response	Early clinical trial	23582881
	PARP inhibitors	Endometrial cancer	Response	Case report	21468130, 20944090
BAP1 Deletion (n=1)	PARP inhibitors	Renal cell carcinoma	Sensitivity	Preclinical	22683710
	HDAC inhibitors	Melanoma	Sensitivity	Preclinical	22038994

The order of presentation of genomic alterations is not necessarily related to the clinical relevance or the potential efficacy of therapies. Additional associations may exist which are not shown because they do not provide different therapeutic options or higher levels of evidence

III

CANCER DRUGS ASSOCIATED TO THE DETECTED GENOMIC ALTERATIONS

- **Everolimus, Temsirolimus:** PTEN, FLCN or PIK3R1 inactivating genomic alterations have been related to response/sensitivity to PI3K/mTOR pathway inhibitors, such as GDC-0941, everolimus or LY3023414, in non small cell lung cancer, prostate cancer, renal cell carcinoma and endometrial cancer. Everolimus is an mTOR inhibitor approved by the FDA (not yet by the EMA) for the treatment of neuroendocrine lung tumors, among other cancer types. Everolimus is approved by the EMA for the treatment of breast cancer, pancreatic neuroendocrine tumors, and renal cell carcinomas. Temsirolimus is another mTOR inhibitor approved for the treatment of renal cell carcinoma, and approved by the EMA (not yet by the FDA) for the treatment of mantle cell lymphoma.
- **Olaparib:** PTEN, BAP1 or BRCA2 inactivating genomic alterations have been related to response/sensitivity to PARP inhibitors, such as olaparib or KU0058948, in endometrial cancer, renal cell carcinoma and ovarian cancer. There are no PARP inhibitors approved for the treatment of neuroendocrine lung tumors. Olaparib is a PARP inhibitor approved for the treatment of ovarian cancer.
- **Valproic acid, Vorinostat, Panobinostat:** BAP1 inactivating genomic alterations have been related to sensitivity to HDAC inhibitors, such as valproic acid, trichostatin A, panobinostat and suberoylanilide hydroxamic acid, in melanoma. There are no HDAC inhibitors approved for the treatment of neuroendocrine lung tumors. Valproic acid is an HDAC inhibitor approved for the treatment of several neurological disorders. Likewise, vorinostat is an HDAC inhibitor approved for the treatment of cutaneous T-cell lymphoma (CTCL), whereas panobinostat is another HDAC inhibitor approved by the EMA (not yet by the FDA) for the treatment of multiple mieloma.

IV

ADDITIONAL COMMENTS

The copy number detected for the tumor suppressor BAP1 (n=1; compatible with the presence of a wild-type allele) together with the lack of detection of additional inactivation alterations of this gene, do not support its putative loss of function in the tumor.

V

TP53 genomic status	Mutated	Alteration	p.Gln317* + Deletion	Alteration type	Frameshift / Premature STOP + Copy number loss (n=1)	% Tum. cel. mut.	100%

TP53 genomic status is shown exclusively as complementary information as, currently, it is not associated with any approved targeted cancer therapy

VI

GENOMIC ALTERATIONS DETECTED WITH UNCERTAIN CLINICAL SIGNIFICANCE — 5 ALTERATIONS

Gene	Alteration	Alteration type	% Tum. cel. mut.
CTNNB1	Deletion	Copy number loss (n=1)	Not applicable
MAP2K1	p.Arg201His	Missense	50%-100%
MITF	Deletion	Copy number loss (n=1)	Not applicable
PTCH1	p.Pro299Leu	Missense	55%-100%
RAF1	Deletion	Copy number loss (n=1)	Not applicable

VII

AVERAGE COVERAGE	% OF COVERED SEQUENCE (CALLABILITY)			
	≥10 READS (DP10) ≥20	READS (DP20) ≥50	READS (DP50) ≥50	READS (DP100)
1201.80x	100%	99.999%	99.91%	99.62%

1 OF THE 1538 GENOMIC REGIONS ANALYZED HAS SHOWN COVERAGE BELOW 100% AT DP20 (20 READS)

Coordinates	Size	% OF SEQUENCE COVERED BY ≥10 READS (DP10)	% OF SEQUENCE COVERED BY ≥20 READS (DP20)	GENE	RefSeq ID	Specific positions with less than 20 reads
2:212488638-212495329	6692	100%	99.985%	ERBB4	NM_005235	c.2079+957

Cabanillas *et al.* Figure S1
