# Supplementary material for: A novel molecular diagnostics platform for somatic and germline precision oncology
Source: Mol Genet Genomic Med. 2017 Apr 23;5(4):336–59. doi: 10.1002/mgg3.291 (PMC5511795; doi:10.1002/mgg3.291)
Supplement: Supplementary file 2 — Figure S2. Front page of the germline sample report. [file MGG3-5-336-s002.pdf]

Sample Germline Report – Front Page

PATHOGENIC VARIANTS

Gene	Variant		Alteration type	Zygosity	Cancer phenotypes associated to the variant
	DNA	Protein			
NF1	c.2033dupC	p.I679Dfs*21	Frameshift/ Premature STOP	Mosaic*	Neurofibromatosis, Type 1

**The frequency of this variant in the analyzed sample is 0.103. This is compatible with a mosaic mutation present in heterozygosity in 20.6% of nucleated blood cells (primary sample from which germline DNA was obtained)*

LIKELY PATHOGENIC VARIANTS

No variant of this category has been found

VARIANTS WITH UNCERTAIN CLINICAL SIGNIFICANCE

Gene	Variant		Alteration type	Zygosity	Cancer phenotypes associated to the gene
	DNA	Protein			
BRCA1	c.3083G>A	p.R1028H	Missense	Heterozygous	Breast and/or ovary cancer
FANCA	c.3348+18A>G	No aplicable	5' UTR	Heterozygous	Fancony anemia (autosomal recessive inheritance) Association with breast cancer

ADDITIONAL COMMENTS

Regarding the reason for request, the analysis of genetic alterations in MLH1, MSH2, MSH6, PMS2 and EPCAM has not revealed alterations considered pathogenic.

The pathogenic variant NF1 p.I679Dfs*21 has been validated by PCR + Sanger sequencing, obtaining results consistent with its presence in the indicated frequency (0.103), what is compatible with the patient being a mosaic for this alteration.

The clinical consequences of the results herein reported must be evaluated by a genetic counsellor.

Cabanillas *et al.* Figure S2
