# Supplementary material for: A novel molecular diagnostics platform for somatic and germline precision oncology
Source: Mol Genet Genomic Med. 2017 Apr 23;5(4):336–59. doi: 10.1002/mgg3.291 (PMC5511795; doi:10.1002/mgg3.291)
Supplement: Supplementary file 3 — Figure S3. Detailed information on germline variants. [file MGG3-5-336-s003.pdf]

Sample Germline Report – Variant Details

DETAILED INFORMATION ON THE PATHOGENIC VARIANTS

NF1

Variant		RefSeq ID	Alteration type	Zygosity	Variant associated phenotypes
DNA	Protein				
c.2033dupC	p.I679fs*21	NM_000267	Frameshift/ Premature STOP	Mosaic*	Neurofibromatosis, Type 1

OTHER CANCER PHENOTYPES ASSOCIATED TO THE GENE

None

VARIANT ASSOCIATED REFERENCES

PMID	Type	Notes
7655472	Analysis of the NF1 gene in patients with truncated NF1 proteins	(1995) Variant identified in 2/14 NF1 patients with NF1 truncated proteins

FEATURES OF THE GENOMIC VARIANT

Genomic coordinates	Reference genotype	Detected genotype
17:29553477	A/A	A/AC
**Coverage**	**dbSNP ID**	**Max. allele frequency (database; allele number)**
1374	rs780157990, rs587781807	<0.00006 (ExAC; 7/120466)

**The frequency of this variant in the analyzed sample is 0.103. This is compatible with a mosaic variant which would be present in heterozygosis in 20.6% of nucleated blood cells (primary sample from which the analyzed DNA was obtained)*

DETAILED INFORMATION ON THE VARIANTS OF UNCERTAIN CLINICAL SIGNIFICANCE

BRCA1

Variant		RefSeq ID	Type of alteration	Zygosity	Cancer phenotypes associated to the gene
DNA	Protein				
c.3083G>A	p.R1028H	NM_007294	Non synonymous	Heterozygosis	Breast and/or ovarian cancer

CATEGORY

ABSENT: variants not present in HGMD, without predicted drastic effects on the structure or the expression of the protein or gene, affecting any of the genes analyzed (see technical data)

CANCER PHENOTYPES AND FUNCTIONAL ALTERATIONS ASSOCIATED TO THE VARIANT

None

VARIANT ASSOCIATED REFERENCES

PMID	Type	Notes
17924331	*In silico* analysis of pathogenicity	100:1 likelihood in favour of neutrality
21990134	Multifactorial analysis of pathogenicity	Likelihood of pathogenicity lower than 9.98 x 10^{-6}

FUNCTIONAL EVALUATION	EVOLUTIVE CONSERVATION OF AFFECTED NUCLEOTIDE
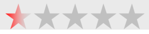	Not conserved

FEATURES OF THE GENOMIC VARIANT

Genomic coordinates	Reference genotype	Detected genotype
17:41244465	C/C	C/T
**Coverage**	**dbSNP ID**	**Max. allele frequency (database; allele number)**
1273	rs80357459	0.00080 (1000 Genomes; 4/5008)

Cabanillas *et al.* Figure S3
