# Supplementary figures and images for: A novel molecular diagnostics platform for somatic and germline precision oncology
Source: Mol Genet Genomic Med. 2017 Apr 23;5(4):336–59. doi: 10.1002/mgg3.291 (PMC5511795; doi:10.1002/mgg3.291)

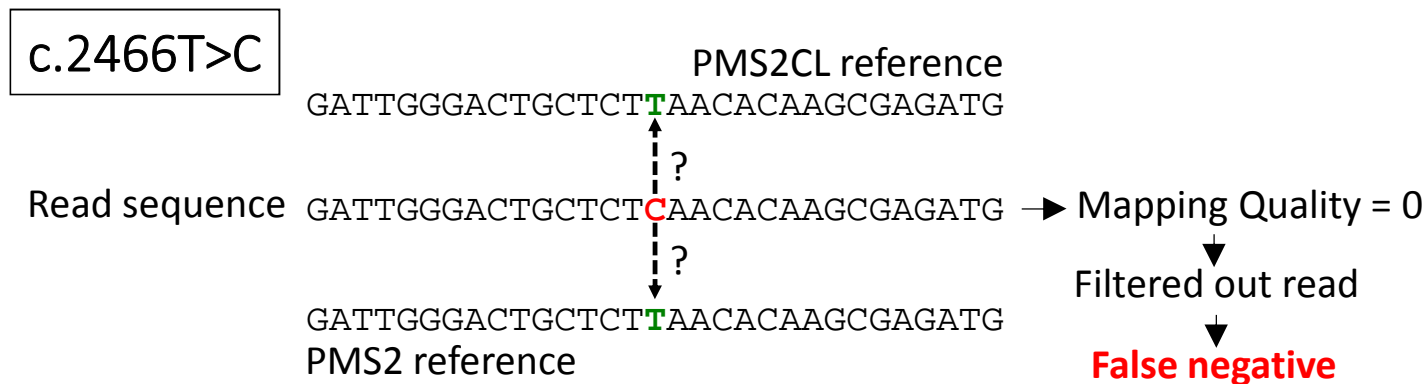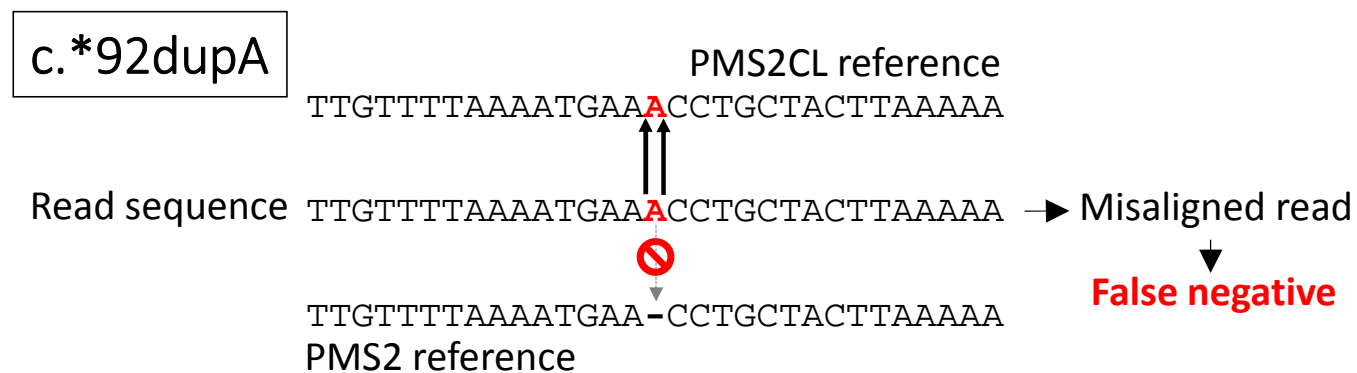

Supplement: Supplementary file 4 — Figure S4. Causes of false negative calling of variants in highly homologous PMS2 regions. [file MGG3-5-336-s004.pdf]
