# Supplementary material for: A novel molecular diagnostics platform for somatic and germline precision oncology
Source: Mol Genet Genomic Med. 2017 Apr 23;5(4):336–59. doi: 10.1002/mgg3.291 (PMC5511795; doi:10.1002/mgg3.291)
Supplement: Supplementary file 5 — Figure S5. Sanger validation of the mosaic NF1 c.2033dupC mutation. [file MGG3-5-336-s005.pdf]

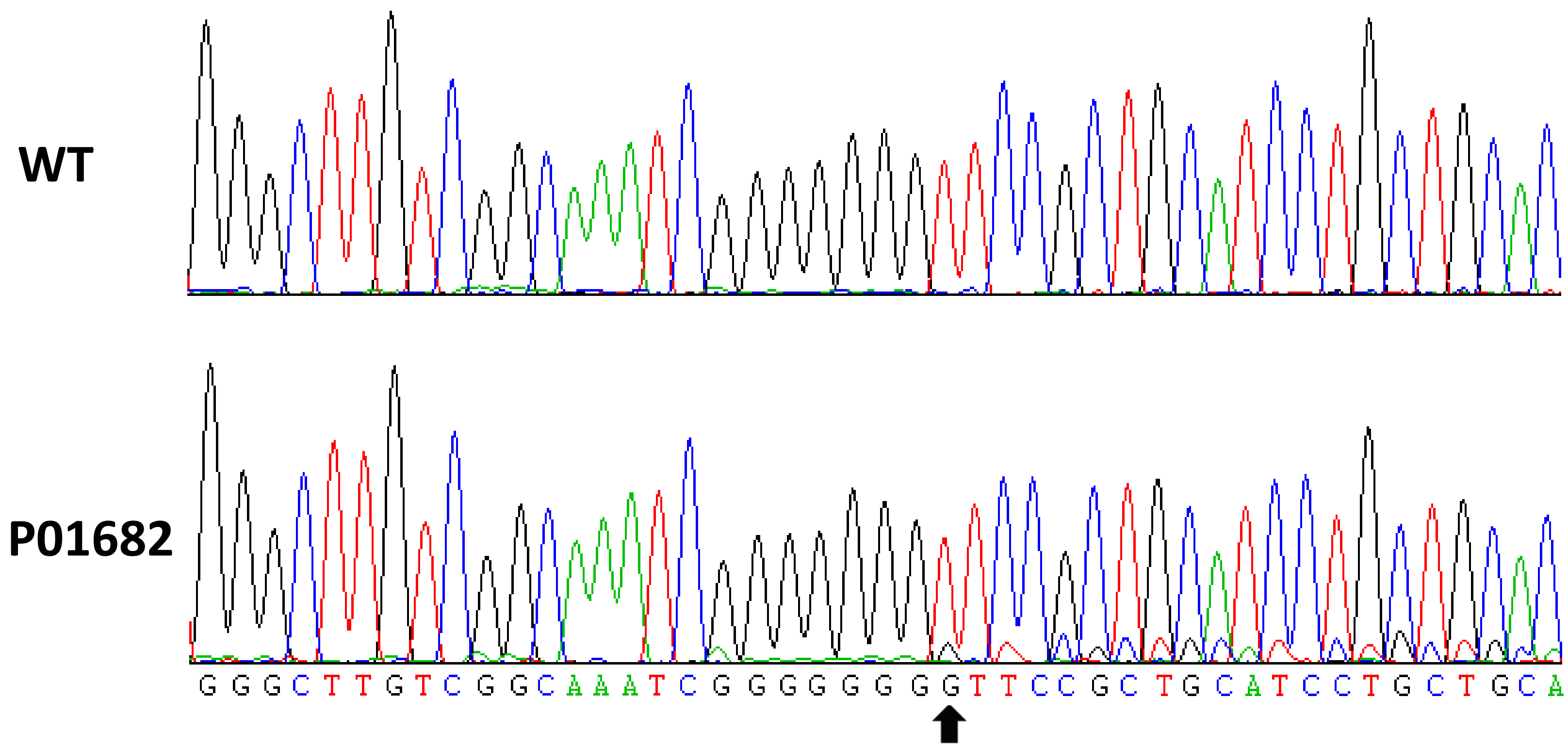

Mosaic NF1 (NM_000267) c.2033dupC, p.(I679Dfs*21) (10.3%)

Cabanillas *et al.* Figure S5
